# Injectable ultrasound-powered bone-adhesive nanocomposite hydrogel for electrically accelerated irregular bone defect healing

**DOI:** 10.1186/s12951-024-02320-y

**Published:** 2024-02-07

**Authors:** Shiqi Zhou, Cairong Xiao, Lei Fan, Jinghong Yang, Ruihan Ge, Min Cai, Kaiting Yuan, Changhao Li, Ross William Crawford, Yin Xiao, Peng Yu, Chunlin Deng, Chengyun Ning, Lei Zhou, Yan Wang

**Affiliations:** 1 Hospital of Stomatology, Guanghua School of Stomatology, Guangdong Provincial Key Laboratory of Stomatology, Sun Yat-sen University, Guangzhou, Guangdong 510055 China; 2 School of Materials Science and Engineering, National Engineering Research Center for Tissue Restoration and Reconstruction, South China University of Technology, Guangzhou, Guangdong 510641 China; 3 Guangzhou Key Laboratory of Spine Disease Prevention and Treatment, Department of Spine Surgery, The Third Affiliated Hospital, Guangzhou Medical University, Guangzhou, Guangdong 510150 China; 4 Department of Orthopedic Surgery, Nanfang Hospital, Southern Medical University, Guangzhou, Guangdong 510515 China; 5 Institute of Health and Biomedical Innovation & Australia-China Centre for Tissue Engineering and Regenerative Medicine, Centre for Biomedical Technologies, Queensland University of Technology, Queensland, 4059 Australia; 6 School of Medicine and Dentistry & Menzies Health Institute Queensland, Griffith University, Queensland, 4111 Australia

**Keywords:** Nanocomposite hydrogel, Bone adhesive, Injectability and self-healing, Bone defects, Electrical stimulation

## Abstract

The treatment of critical-size bone defects with irregular shapes remains a major challenge in the field of orthopedics. Bone implants with adaptability to complex morphological bone defects, bone-adhesive properties, and potent osteogenic capacity are necessary. Here, a shape-adaptive, highly bone-adhesive, and ultrasound-powered injectable nanocomposite hydrogel is developed via dynamic covalent crosslinking of amine-modified piezoelectric nanoparticles and biopolymer hydrogel networks for electrically accelerated bone healing. Depending on the inorganic-organic interaction between the amino-modified piezoelectric nanoparticles and the bio-adhesive hydrogel network, the bone adhesive strength of the prepared hydrogel exhibited an approximately 3-fold increase. In response to ultrasound radiation, the nanocomposite hydrogel could generate a controllable electrical output (-41.16 to 61.82 mV) to enhance the osteogenic effect in vitro and in vivo significantly. Rat critical-size calvarial defect repair validates accelerated bone healing. In addition, bioinformatics analysis reveals that the ultrasound-responsive nanocomposite hydrogel enhanced the osteogenic differentiation of bone mesenchymal stem cells by increasing calcium ion influx and up-regulating the PI3K/AKT and MEK/ERK signaling pathways. Overall, the present work reveals a novel wireless ultrasound-powered bone-adhesive nanocomposite hydrogel that broadens the therapeutic horizons for irregular bone defects.

**Supplementary Information:**

The online version contains supplementary material available at 10.1186/s12951-024-02320-y.

## Introduction

Bone defects caused by traffic accidents, bone tumors, or specific diseases (such as chronic inflammation and necrosis) impose significant physical and psychological challenges on patients [1–3]. Reconstruction and repair of critical bone defects remain challenging, particularly in cases of deep bone defects with irregular shapes [4, 5]. Autologous bone grafting is currently the gold standard for bone repair [6]. However, its clinical application is severely limited due to low availability, high donor site morbidity, and inadequate adaptability to irregular defects [7, 8]. In recent years, researchers have made numerous attempts to enhance orthopedic implants for the treatment of complex bone defects [9–11]. For instance, surface modification of implants to incorporate osteogenic differentiation factors or the design of 3D porous structures for the delivery of osteogenic drugs [12, 13]. However, these approaches are hindered by limited delivery efficiency and the potential for drug inactivation. In contrast to traditional methods that focus on surface functional group engineering and biochemical molecule delivery, simulating the extracellular matrix microenvironment offers a more direct and effective strategy to regulate osteogenesis [14, 15]. It has been discovered that altering the hydrophilicity of materials can modulate the behavior of bone marrow mesenchymal stem cells (BMSCs) and promote osteogenic differentiation [13, 16, 17]. Moreover, material stiffness can induce the expression of mechanosensitive proteins, the secretion of pro-angiogenic molecules, and accelerate bone healing [11, 18–20]. Therefore, endowing implant materials with biophysical cues that mimic extracellular matrix is a feasible approach to treating irregularly shaped bone defects.

As a biophysical stimulus, bioelectrical signals are prevalent in the microenvironment of native bone tissue and play a vital role in facilitating the bone defect healing process [21–23]. Researchers have observed the presence of electrical potentials in natural bone and periosteum [24–26]. Moreover, following the occurrence of a fracture, an endogenous negative potential emerges at the site of injury, directly influencing cell proliferation and differentiation and thereby mediating bone repair [21, 27, 28]. Increasing evidence suggests that bionic exogenous electrical stimulation can regulate bioelectrical signals and accelerate bone defect healing. Exogenous electrical stimulation therapy, approved by the US Food and Drug Administration, has demonstrated its capacity to accelerate bone defect healing [29]. A pulsed electric field of appropriate intensity and frequency can activate the expression of osteogenesis-related genes, promote the proliferation and differentiation of tissue cells at the defect, and actively induce bone regeneration [30]. However, clinically available electrical stimulation therapy relies on large and complex equipment, and long-term treatment is prone to skin inflammation, which limits the clinical application of electrical stimulation therapy [31]. Alternatively, wireless-powered stimulation bone implant materials present a promising solution to address these challenges, avoiding wiring problems and improving efficacy [32]. 

Piezoelectrically bioactive materials, which generate physiologically relevant electrical signals in response to ultrasound or acoustic power (direct piezoelectric effect), hold great promise for delivering stable electrical stimulation [33, 34]. Extensive research has demonstrated that piezoelectric ceramics such as barium titanate, potassium niobate, and strontium titanate can generate electrical stimulation through voltage polarization or mechanical force [35, 36]. This stimulation has been shown to promote the migration of key cells involved in bone defect healing, increase the expression of osteogenic differentiation factors, and accelerate bone defect repair [27, 37]. However, the high brittleness and poor flexibility of piezoceramics hinder their adaptation to irregularly shaped fracture sites, significantly limiting their effectiveness in electrical stimulation regeneration. On the other hand, flexible and easily processable PVDF polymer films have shown the ability to generate electrical stimulation and induce neural differentiation of BMSCs [38, 39]. Unfortunately, these piezoelectric implantable scaffolds lack bone-adhesive properties and cannot be used to treat highly comminuted fractures that require initial fixation. Moreover, poor bone adhesion might allow material to move around complex bone defect sites, causing inflammation or damage to surrounding tissues [40, 41]. Implant materials also lack shape adaptability and adhesive properties, resulting in poor contact with the host bone defect area, further promoting bone resorption and impeding osseointegration [8]. Therefore, we hypothesized that a novel bone implant material that simultaneously achieves self-adaptation, bone adhesion, and ultrasound powering could accelerate irregular bone defect healing.

**Scheme 1:**
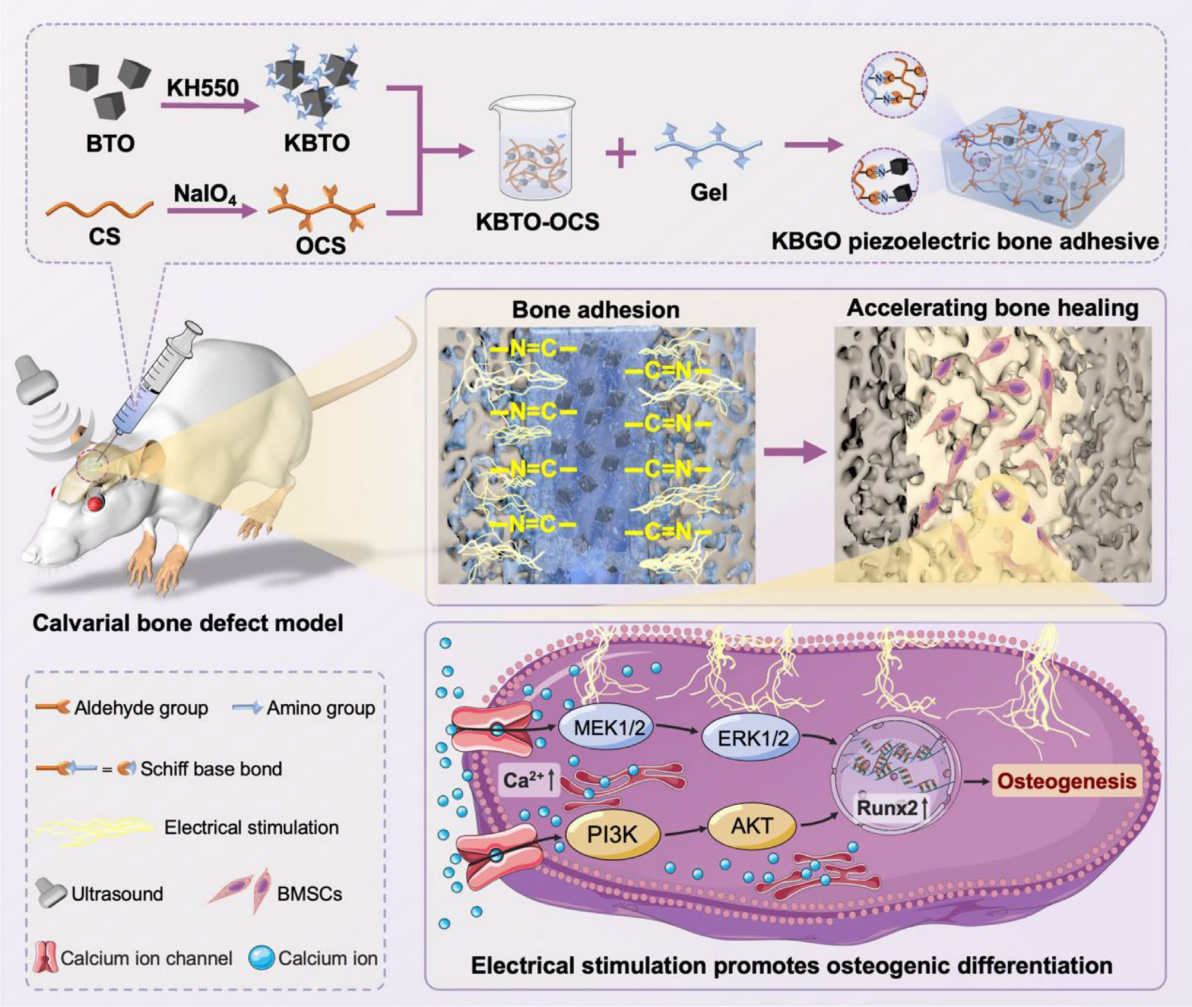
Schematic diagram illustrating the synthesis of an injectable ultrasound-powered bone-adhesive nanocomposite hydrogel and its application in electrically accelerated bone defect healing

Herein, we constructed an injectable ultrasound-powered bone-adhesive hydrogel for irregular bone defect repair (Scheme 1). The nanocomposite hydrogel is composed of functionalized piezoelectric nanoparticles and a dynamically cross-linked natural polymer hydrogel, which exhibits injectable, self-adaptive, and bone-adhesive properties. Based on the inorganic-in-organic integration between amino-modified barium titanate nanoparticles and bio-adhesive gelatin-chondroitin sulfate networks, the mechanical properties of these novel hydrogels could be enhanced, thereby promoting the adhesive effect of these hydrogels to bone tissues. More significantly, benefiting from the incorporation of piezoelectric nanoparticles, the obtained hydrogel system could effectively generate electrical stimulation to promote cell osteogenic differentiation in vitro under ultrasound irradiation. The results of the in vivo bone defect healing test and histopathological assessment proved that the electrical stimulation generated by piezoelectric hydrogel could accelerate the healing of calvarial bone defects. In addition, we investigated the mechanism underlying the stimulatory effect of electrical stimulation generated by ultrasound-powered piezoelectric hydrogel on bone healing. This study highlights the potential of ultrasound-powered piezoelectric hydrogel with self-adaptive and highly adhesive capabilities in bone tissue engineering and provides a novel strategy for treating irregular bone defects.

## Materials and methods

### Fabrication of injectable ultrasound-powered nanocomposite hydrogels

The hydrothermal method was used to produce barium titanate (BaTiO_3_, BTO) nanoparticles using Ba(OH)_2_·8H_2_O as the barium source and tetrabutyl titanate as the titanium source [42, 43]. By grafting amino groups (-NH_2_) onto BTO particles (KBTO), a silane coupling agent (3-Aminopropyltriethoxysilane, KH550; A3648, Sigma-Aldrich, America) was employed. 5% m/v of oxidized chondroitin sulfate (OCS) was weighed and dissolved in a PBS solution containing 0.05 M borax and then heated in a 37 °C water bath for 5 min to dissolve completely. Then 0%, 0.1%, and 0.5% m/v KBTO nanoparticles—corresponding to GO, 0.1KBGO, and 0.5KBGO hydrogels—were added and shaken for 5 min in an ultrasonic cleaner to speed up the dissolution. 10% m/v gelatin (Gel) was added after the nanoparticles had been distributed evenly, and the mixture was then put in a 37 °C water bath with magnetic stirring to speed up the dissolution process. The injectable ultrasound-powered nanocomposite hydrogels were created through Schiff base bonds and hydrogen bonds.

### Characterization of ultrasound-powered piezoelectric hydrogels

The surface morphologies, crystal structures, particle sizes, and chemical bonds of KBTO nanoparticles were characterized by scanning electron microscopy (SEM; Merlin, Carl Zeiss, Germany), transmission electron microscopy (TEM; JEM-2100 F, JEOL, Japan), X-ray diffractometer (XRD; D8 advance, Bruker, Germany), dynamic laser scatterer (DLS; Zetasizer Nano ZS, Malvern, UK), atomic force microscopy (AFM; Multimode 8, Bruker, Germany), and Fourier transform infrared spectrometer (FTIR; Nicolet6700, Thermo Fisher, USA). Moreover, the potential distribution of KBTO nanoparticles under stress was simulated by finite-element simulation with COMSOL.

A nuclear magnetic resonance spectrometer (NMR; AVANCE III HD 400, Bruker, Switzerland) was used to ascertain the successful grafting of aldehyde groups onto OCS. In a concise procedure, 10 mg each of CS and OCS were completely dissolved in 0.6 mL of deuterated water (D_2_O) and subsequently transferred to a quartz NMR tube. The ^1^H NMR spectra of OCS and CS were acquired, with tetramethylsilane (TMS) employed as the internal standard. Then, FTIR, XRD, and EDS were used to characterize the crystalline properties, interface structures, and chemical compositions of ultrasound-powered nanocomposite hydrogels, and the surface morphologies of piezoelectric hydrogels were observed by SEM. In addition, to visualize the distribution of KBTO nanoparticles in the piezoelectric hydrogel, a confocal laser scanning microscope (CLSM; TCS SP8, Leica, Germany) was applied to observe the incorporated nanoparticles labeled with Rhodamine B dye (R104960, Aladdin, China).

### Rheological behavior analysis and macroscopic self-healing experiment

A rotational rheometer (MCR301, Anton Paar, Austria) was used to evaluate the gelation time, shear thinning properties, and self-healing behaviors of the hydrogels. Generally, an oscillation time sweep with 1 Hz oscillation frequency and 5% strain amplitude was exerted to obtain the storage modulus (G′) and the loss modulus (G”) to determine the gelation time and characterize the rheological behavior of hydrogels during gelation. Hydrogel cylinders were prepared (Φ 20 mm × 5 mm), then the frequency-modulus curves of angular frequencies ranging from 0.1 to 100 rad/s were recorded at 5% strain amplitude. The relationships between linear viscosity and shear rate were examined in a frequency sweep mode to access the shear-thinning behavior of piezoelectric hydrogels.

In order to validate the self-healing properties of the hydrogel, a strain amplitude sweep test was conducted at a consistent frequency of 10 rad/s at 37 °C, employing a strain range spanning from 0.1 to 1000%. This test aimed to elucidate the critical point at which the hydrogel network structure undergoes collapse. Then an alternate strain sweep test was carried out at a fixed angular frequency of 10 rad/s at 37 °C. And the oscillatory strains were alternated between a small strain (5%) and a large strain (1000%) for five cycles, with a duration of 200 s for each strain value. Macroscopic self-healing experiments provided an intuitive demonstration of the self-healing behavior of the hydrogel. Two same hydrogel disks (Φ 20 mm × 3 mm) were prepared, with one of them was stained red using Rhodamine B dye. Subsequently, the two hydrogel disks were symmetrically cut into two pieces, which were connected with the exchanged pieces at 37 °C. The self-healing time was recorded. The reconnected hydrogel pieces were then picked up with a tweezer and pulled to observe the macroscopic self-healing ability. Furthermore, a dynamic mechanical analyzer (DMA Q800, TAInstruments, USA) was employed to assess the mechanical properties of the hydrogel both before and after the healing process. The samples, configured as elongated strips with dimensions of 30 mm in length, 10 mm in width, and 2 mm in thickness, were subjected to stretching using a clamp at a force rate of 0.5 N/min, and the original stress-strain curves were recorded. Subsequently, the samples were bisected and rejoined at 37 °C until complete healing occurred. Following the healing period, they were once again stretched at a force rate of 0.5 N/min, and the resulting stress-strain curve was recorded.

### In vitro adhesion experiments on porcine femur

To quantify the adhesion properties of ultrasound-powered nanocomposite hydrogel to bone tissue, in vitro adhesion experiments were carried out on a fresh porcine femur. Briefly, 50 µL hydrogel was injected on the cancellous bone fragment surfaces sawn from the femur, which were then adhered together in an end-to-end and lap-shear manner. After the hydrogel was completely solidified for 5 min at 37 °C, tensile mechanical tests were carried out by a universal material testing machine (Z 100, Zwick Roell, Germany) at a rate of 2 mm/min in both end-to-end and lap-shear directions to obtain representative stress-displacement curves and record the maximum stress per unit area before adhesion failure.

### Detections of the output voltages of ultrasound-powered nanocomposite hydrogels

The piezoelectric voltage output test system consisted of ultrasonic equipment, a high-precision general-purpose digital instrument (DMM7510, Keithley, USA), and a computer. The cylindrical hydrogels were prepared with a diameter of 10 mm and a thickness of 2 mm (Φ10 mm × 2 mm). Round copper sheets (Φ10 mm) were used as electrodes on the top and bottom surfaces of the hydrogels. Then the hydrogels and copper sheets were completely immersed into a container with water, and it was made sure that there was 10 cm of space between the hydrogel and the ultrasound probe. Then the output voltages of the ultrasound-powered nanocomposite hydrogels under different ultrasonic sound intensities and with different doping concentrations of KBTO nanoparticles and BTO nanoparticles were investigated.

### Cell culture

Bone mesenchymal stem cells (BMSCs) were harvested from C57BL/6 aged 3–5 weeks. Briefly, the bone medullary cavities of the isolated tibia and femur were repeatedly flushed with α-MEM (C11095500BT, GIBCO, America). Then the bone marrow suspension was filtered using a 70 μm cell strainer (431,751, Corning, America) and centrifuged at 1000 rpm for 5 min. The cells were cultured in α-MEM supplemented with 10% fetal bovine serum (FBS; 10,099,141 C, GIBCO, America) and 1% Penicillin-Streptomycin (15,140,122, GIBCO, America) at 37 °C and 5% CO_2_. The following experiments employed the cells at passages 3 to 5.

### Cell viability and proliferation assays

There are six groups: GO (GO hydrogel), GO + US (GO hydrogel and ultrasound cavitation), 0.1KBGO (0.1KBGO hydrogel), 0.1KBGO + US (0.1KBGO hydrogel and ultrasound cavitation), 0.5KBGO (0.5KBGO hydrogel), and 0.5KBGO + US (0.5KBGO hydrogel and ultrasound cavitation). The GO, 0.1KBGO, and 0.5KBGO hydrogels were added to 48-well plates. Then BMSCs were seeded in each well at a density of 2 × 10^4^. After that, the US groups received ultrasonic loading with a sound intensity of 1.5 W/cm^2^ for 5 min per day, while other groups were left untreated. After 48 h, a Calcein-AM/PI Live/Dead assay kit (BB-4126, Bestbio, China) was used to monitor the viability of the seeded BMSCs under a CLSM. After 1, 4, and 7 days, a Cell Counting Kit-8 (CCK-8; Dojindo, Kumamoto, Japan) was applied to evaluate the proliferation of BMSCs on different hydrogels, and the optical density (OD) was detected by a microplate spectrophotometer (Epoch2, BioTek, USA) at 450 nm. And the cell nuclei and cytoskeleton of BMSCs were stained by DAPI staining solution (C1006, Beyotime, China) and actin-tracker green (C2201S, Beyotime, China) and then visualized by a CLSM to evaluate the cell spreading and adhesion after being cultured for 48 h.

### Hemolysis test

To evaluate the hemocompatibility of hydrogels, a total of six groups were set up, namely the Triton, GO, 0.1KBGO, 0.5KBGO, and PBS groups, in which the PBS was set as a negative control and 0.1% Triton X-100 was utilized as a positive control. 1 ml of whole blood was obtained from rats to incubate with each group for 2 h at 37 °C. After centrifuging (at 1000 rpm) each sample for 10 min at 4 °C, the 100 µL supernatant was transferred to a 96-well plate to detect the OD value with a microplate spectrophotometer at 540 nm.

### Evaluation of the osteogenic assay of ultrasound-powered piezoelectric hydrogels in vitro

There are six groups: GO, GO + US, 0.1KBGO, 0.1KBGO + US, 0.5KBGO, and 0.5KBGO + US. These hydrogels were added to 48-well plates. Then BMSCs were seeded in each well at a density of 2 × 10^4^ and incubated in an osteoblastic induction medium refreshed every second day. Among them, the US groups were subjected to ultrasonic loading with a sound intensity of 1.5 W/cm^2^ for 5 min every day, and the rest were left untreated. After 7 and 14 days, an ALP kit (A059-3-1, Nanjing, China) was used to detect the early osteogenic differentiation marker alkaline phosphatase (ALP) activity, and then a bicinchoninic acid (BCA) kit for protein determination (P0010, Beyotime, China) was applied to normalize it to the total protein, and the ALP staining was performed. The ALP expression was observed under a stereoscopic microscope (MZ10F, Leica, Germany). To evaluate the effects of piezoelectric stimulation on proteins and genes related to osteogenic differentiation of BMSCs. RT-qPCR detection was performed on both days 7 and 14 to evaluate the expression of osteogenic-related genes ALP, runt-related transcription factor 2 (Runx2), bone morphogenetic protein 2 (BMP2), osteopontin (OPN), osteocalcin (OCN), and collagen I (COL- I). All primer sequences used in RT-qPCR were presented in Table S1. In addition, immunofluorescence staining of Runx2 and OCN was also performed on day 7 [44]. After being counterstained with actin-tracker green and DAPI, BMSCs were observed under CLSM. Western blot was carried out to assessed the expression levels of proteins in PI3K/AKT and MEK/ERK signaling pathways as well as markers of osteogenic differentiation [45]. Primary and secondary antibodies were displayed in Table S2.

### Evaluation of in vivo degradation and biocompatibility of piezoelectric hydrogels

Eight adult male Sprague Dawley (SD) rats (300 ~ 350 g) were assigned to two groups at random, group GO and 0.1KBGO, to investigate the degradation of piezoelectric hydrogels in vivo. The hydrogels were implanted subcutaneously in the rats’ back. After 2 and 6 weeks, the rats were euthanized via carbon dioxide asphyxiation and cervical dislocation. The tissues at the implantation sites were excised and stained with hematoxylin and eosin (H&E; C0105S, Beyotime, China) to observe the inflammatory reaction induced by the hydrogels.

As for the assessment of the in vivo histocompatibility of hydrogels, sixteen rats were randomly assigned to four groups: group GO, GO + US, 0.1KBGO, and 0.1KBGO + US. Rat serum was collected at week 12 to detect the levels of several biochemical indicators, such as aspartate aminotransferase (AST), alanine aminotransferase (ALT), and total protein (TP) levels, which help assess the systemic toxicity of hydrogels. At the same time, after the rats in four groups were euthanized, their heart, liver, spleen, lung, and kidney tissues were harvested and fixed in 4% PFA for embedding, slicing, and H&E staining. The accumulation of hydrogel degradation products and pathological changes were observed in these tissue sections under a microscope.

### Repair assay in the rat critical-sized calvarial defect model

This experiment was approved by the Animal Ethics Committee of South China University of Technology with approval number AEC2021068. Thirty-two adult male SD rats (300 ~ 350 g) were assigned to four groups at random, group GO, GO + US, 0.1KBGO, and 0.1KBGO + US. To anesthetize the rats, 3% sodium pentobarbital (50 mg/kg) was given intraperitoneally. Then the operation region hair was shaved and sterilized with 0.5% iodine volts. The skull bone plate was exposed in a sterile environment by bluntly dissecting the skin and periosteum. Then a critical-size bone defect area (d = 5 mm) at a distance of about 1 mm from both sides of the calvarial raphe was drilled with sterile normal saline to cool down. After each hydrogel was injected into the prepared bone defect area, the incision was sutured in layers. The rats were housed in separate cages at 23 ± 3 °C and given free access to food. The US groups underwent ultrasound loading with a sound intensity of 1.5 W/cm^2^ for 5 min daily, commencing three days post-surgery. Conversely, the other groups received no ultrasound loading. At 6 and 12 weeks, the rats were euthanized via carbon dioxide asphyxiation and cervical dislocation. Then the calvaria were completely separated and fixed in 4% PFA for further detection. A micro-computed tomography (Micro-CT; µCT 100, Scanco Medicine AG, Switzerland) was applied to measure a variety of bone parameters, including bone mineral density (BMD), bone volume/total volume (BV/TV), trabecular number (Tb.N), and trabecular thickness (Tb.Th), to assess the in vivo reparative effectiveness of the ultrasound-powered nanocomposite hydrogels. Moreover, the calvaria were decalcified with 10% EDTA, dehydrated with ethanol gradients, embedded in paraffin, and sliced into coronal sections for H&E staining, Masson’s Trichome (MT; G1340, Solarbio, China) staining, and immunocytochemistry staining of Runx2 and OCN, respectively.

### Transcriptome sequencing

There are four groups: GO, GO + US, 0.1KBGO, and 0.1KBGO + US. Two milliliters of BMSCs suspension (1 × 10^5^) were seeded on different samples in six-well plates. Among them, the US groups were subjected to the stress generated by an ultrasonic sound intensity of 1.5 W/cm^2^ for 5 min per day, and others were left untreated. Then total RNA extracted with TRIzol reagent (15596-026, Invitrogen, USA) was assessed by Nanodrop 2000 Spectrophotometers (ND-2000, Thermo Fisher, USA) and Agilent 2100 Bioanalyzer (Agilent 2100, Agilent, USA) for concentration, purity, and integrity. Once the RNA concentration was ≥ 50 ng/ µL and OD260/OD280 was between 1.8 and 2.2, an Illumina NovaSeq 6000 platform (NovaSeq 6000, Illumina, USA) was used for RNA sequencing. Contaminating reads were eliminated using Cutadapt software, and clean reads were mapped to the genome using HISTA2 software and assembled by StringTie software. A comprehensive transcriptome was merged and established through GFFcompare software. EdgeR was applied to analyze the differential expression of genes. Fragments per kilobase of exon per million mapped fragment reads (FPKM) values were calculated according to the gene lengths and the read counts mapped to the genes, which were used to express relative gene abundance. Gene stability and sensitivity were examined by assessing the consistency of expression among different groups. Furthermore, read counts were normalized, and differentially expressed genes (DEGs) were determined with *p* < 0.05 and | log2 FC (fold change) | ≥ 1. Then the Gene Ontology (GO) and Kyoto Encyclopedia of Genes and Genomes (KEGG) enrichment analyses were carried out on the OmicStudio Cloud Platform (www.omstudio.cn).

### Detection of cell membrane potential and intracellular calcium ion concentration

The assay of cell membrane potential referred to the published literature [46]. DiBAC4(3) was purchased from BestBio (BB-4110, China). The fluorescence images were captured by CLSM after co-culturing cells with the fluorescent probe. And ImageJ software was carried out to measure fluorescence intensity. Fluo-4 acetoxymethyl ester (Fluo-4 AM; S1060, Beyotime, China) is commonly used for the detection of intracellular calcium ions (Ca^2+^) concentration as a fluorescent probe. Fluo-4 produced by the enzymatic hydrolysis of Fluo-4 AM can combine with Ca^2+^ to generate green fluorescence. In brief, BMSCs were cultured on different samples for 24 h and then incubated in a medium containing 2.5 mmol/L Fluo-4 AM for 1 h. On the other hand, we also studied the changes in intracellular Ca^2+^ concentration after Ca^2+^ channels blockade. Gadolinium trichloride (GdCl_3_; G119221, Aladdin, China) as a chelator of Ca^2+^ channels in cell membranes, including voltage-gated and mechanosensitive Ca^2+^ channels, can inhibit Ca^2+^ influx. Briefly, after being incubated for 24 h in a medium containing 10 µmol/L GdCl_3_ on different samples, BMSCs were cultured for 1 h in a medium containing 2.5 mmol/L Fluo-4 AM. Before observation, the US group received ultrasonic loading for 5 min. Subsequently, a CLSM was used to capture fluorescence images in different groups with an excitation wavelength of 488 nm, which were analyzed by ImageJ software.

### Statistical analysis

All data were presented as the mean ± standard deviation (SD) of at least three independent experiments. SPSS 22.0 statistical software was carried out for statistical analysis, and one-way analysis of variance (ANOVA) followed by Tukey’s post hoc test was performed to determine the statistically significant differences (*p*), with a *p*-value < 0.05 deemed significant.

## Results and discussion

### Synthesis and characterization of functionalized barium titanate nanoparticles

It is well-established that tetragonal barium titanate exhibits a non-centrosymmetric spontaneous polarization structure because the centers of the Ti^4+^ cation and O^2−^ anion do not coincide in the unit cell [47]. Therefore, the tetragonal phase structure induces relative displacement of positive and negative charges in response to mechanical strain, ultimately enhancing its piezoelectric potential [48]. To prepare barium titanate (BTO) nanoparticles with mechano-electric response properties, BTO nanoparticles with tetragonal phase structure were prepared by a classical hydrothermal method [34]. The cube-shaped BTO nanoparticles were observed under scanning electron microscopy (SEM) and transmission electron microscopy (TEM) (Figure S1a-c, Supporting Information). As demonstrated in the high-resolution TEM (HRTEM) image (Figure S1d, Supporting Information), the lattice fringes of a single nanoparticle were 0.2827 nm, consistent with the d-spacing of the (110) crystal plane of perovskite barium titanate, suggestive of high crystallinity. In addition, energy dispersive spectroscopy (EDS) results showed that Ba, O, and Ti elements were uniformly distributed on the surface of nanoparticles (Figure S1e-h, Supporting Information). The X-ray diffractometer (XRD) pattern (Figure S2, Supporting Information) showed a distinct splitting peak around 2θ = 45°, and all diffraction peaks were consistent with tetragonal piezoelectric barium titanate (JCPDS data No. 05–0626). Furthermore, the dynamic laser scatterer (DLS) results (Figure S3a, Supporting Information) showed an average particle diameter of 150–200 nm, consistent with the results of SEM and TEM. The above results indicated that the successfully prepared tetragonal BTO nanoparticles with uniform particle size were expected to generate electrical stimulation through mechanical-electrical conversion.

### Synthesis and physical characterization of the ultrasound-powered nanocomposite hydrogels

The stability and mechanical qualities of organic networks are frequently destroyed when BTO nanoparticles are physically blended with them. Hence, to address the crosslinking strength of BTO nanoparticles with oxidized chondroitin sulfate/gelatin (OCS/Gel) organic network, an aminosilane coupling agent (KH-550) was used to graft enough amino groups on the surface of BTO nanoparticles. The absorption peaks at 1105 cm^− 1^, 2385 ~ 2925 cm^− 1,^ and 3450 cm^− 1^ in Fourier transform infrared spectrometer (FTIR) spectra (Figure S4, Supporting Information) corresponded to the stretching vibration of Si-O, the symmetric stretching and asymmetric stretching vibrations of -CH_2_ and -CH_3_, and the bending vibration of -NH_2_, respectively. Moreover, the zeta potential (Figure S3b, Supporting Information) of amino-functionalized BTO (KBTO) was about − 23.0 mV, which was higher than that of BTO (-19.2 mV). The above results indicated that amination treatment promoted the dispersibility of KBTO nanoparticles, which contributed to uniform dispersion in the hydrogel system.

**Fig. 1:**
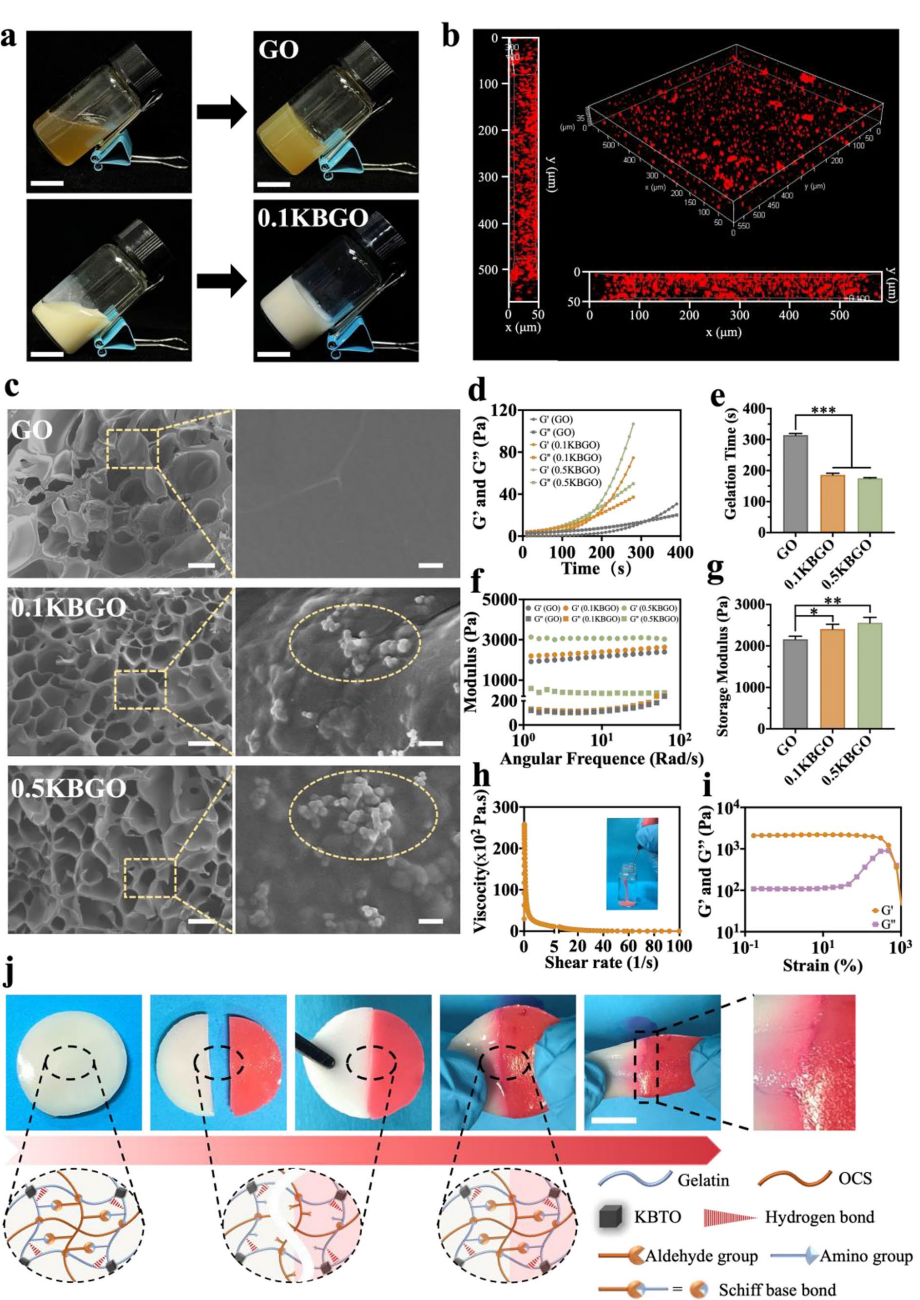
Characteristic of injectable ultrasound-powered nanocomposite hydrogels. (**a**) Optical photos of hydrogels forming. Scale bar represents 1 cm. (**b**) 3D image of the distribution of KBTO nanoparticles (red fluorescence) in the 0.1KBGO hydrogel. (**c**) SEM images of GO, 0.1KBGO, and 0.5KBGO hydrogels and embedded KBTO nanoparticles (dotted bordered ellipse). Scale bars represent 60 µm and 2 µm (higher magnification). (**d**) Oscillation time sweep rheological behaviors of GO, 0.1KBGO, and 0.5KBGO hydrogels. (**e**) Gel time of hydrogels with different KBTO nanoparticles content. (**f**) Storage modulus (G’) and loss modulus (G”) of hydrogels at different shear frequencies. (**g**) Storage modulus at an angular frequency of 10 Rad/s. (**h**) Shear-thinning properties and the injectability image of 0.1KBGO hydrogel. (**i**) The strain amplitude sweep test of 0.1KBGO hydrogel. (**j**) Optical photos and schematic illustration of the self-healing behavior of 0.1KBGO hydrogel. Scale bar represents 1 cm. ANOVA followed by Tukey’s post hoc test was performed for statistical analysis (*p < 0.05, **p < 0.01, ***p < 0.001)

Gel and OCS are widely employed in biomedical fields given their great biocompatibility and adaptability. Therefore, we constructed piezoelectric bone-adhesive nanocomposite hydrogels with Gel and OCS as natural polymer matrix and doped with KBTO nanoparticles, as depicted in Scheme 1. Give that the concentration of KBTO nanoparticles in KBGO hydrogels was 0%, 0.1%, and 0.5% w/v, they were named GO, 0.1KBGO, and 0.5KBGO hydrogel, respectively. Firstly, we used NMR spectroscopy to ascertain the successful oxidation of chondroitin sulfate (CS) to OCS through the application of sodium periodate. The ^1^H NMR spectrum of OCS revealed a distinctive peak at 7.98 ppm, corresponding to the aldehyde group, distinguishing it from the spectrum of CS (Figure S5, Supporting Information). This observation confirms the successful conversion of the hydroxyl groups in CS to aldehyde groups in OCS. As shown in the schematic illustration, the prepolymer solution (OCS/KBTO) was formed by in situ Schiff base reaction of OCS with aldehyde groups and KBTO nanoparticles with amino groups. Macroscopic observation showed that a well-crosslinked and stable hydrogel network structure was constructed through hydrogen bonds and Schiff base bonds formed by Gel and OCS/KBTO (Fig. 1a).

The major part of the FTIR spectra (Figure S6, Supporting Information) of GO, 0.1KBGO, and 0.5KBGO hydrogels was similar. The peaks at around 3520 cm^− 1^, 1637 cm^− 1^, 1540 cm^− 1^, and 1450 cm^− 1^ corresponded to the stretching vibrations of the O-H, the stretching vibrations of the C = O, the bending and stretching vibrations of the N-H, and the in-plane vibration of C-N, respectively, which were characteristic bands of amide bonds. In contrast, the new peaks at 1635 cm^− 1^ and 560 cm^− 1^ represented Ba-Ti-O and Ti-O. Besides, the tetragonal perovskite KBTO structure could be observed in the XRD pattern (Figure S7, Supporting Information) of 0.1KBGO and 0.5KBGO hydrogels. Furthermore, the uniform distribution of KBTO nanoparticles tagged with Rhodamine B dye in piezoelectric hydrogel was visualized by a confocal laser scanning microscope (CLSM) (Fig. 1b), which could be attributed to the homogeneous coupling of amino groups on KBTO with hydroxyl groups on Gel and aldehyde groups on OCS. Next, to characterize the morphology of piezoelectric hydrogels, the cross-sections of the lyophilized hydrogels were examined by SEM (Fig. 1c). These hydrogels had a three-dimensional porous structure, and KBTO nanoparticles were observed in the enlarged images. In addition, the average pore sizes of 0.1KBGO and 0.5KBGO hydrogels were smaller than the GO hydrogel, mainly attributed to the KBTO nanoparticles occupying more cross-linking sites, thus increasing the cross-linking density of 0.1KBGO and 0.5KBGO hydrogels. EDS demonstrated that KBTO nanoparticles were successfully incorporated and uniformly distributed in the hydrogels (Figure S8, Supporting Information). The above results demonstrated that the active dynamic covalent bonds formed by the amino groups on KBTO and the aldehyde groups on OCS were integrated into the organic network, forming a highly integrated inorganic-organic structure.

In the clinical application of bone-adhesive hydrogels, gelation time is an important consideration [49]. An oscillation time sweep was performed on a rheometer to detect the storage modulus (G’) and loss modulus (G’’) of hydrogels over time. The gelation time was determined by the intersection of G’ and G’’ in Fig. 1d. The experiment was repeated 3 times to generate a bar statistic chart (Fig. 1e). In accordance with our anticipated outcomes, the gelation time exhibited a discernible reduction from 314.33 ± 5.13 s to approximately 175.00 ± 2.00 s with the increasing doping concentration of KBTO nanoparticles. This phenomenon can be attributed to the augmented availability of cross-linking sites within the system. Indeed, it is well-established that satisfactory mechanical properties are important for the clinical application of hydrogels for bone defect repair. Gelatin hydrogel is widely recognized for its poor mechanical properties. Over the years, several studies have been conducted to increase mechanical strength by doping inorganic particles [50–52]. In the present study, we hypothesized that the mechanical characteristics of KBTO-incorporated piezoelectric hydrogel could be improved. The frequency sweep rheological test was performed at 37°C. The G’ and G’’ of GO, 0.1KBGO, and 0.5KBGO hydrogels are shown in Fig. 1f. Plateaued moduli differences (G’>G’’) of all groups could be observed throughout the frequency range, demonstrating that the three hydrogels consistently behaved as a stable elastic solid. At the same time, the storage modulus of the hydrogel increased as the concentration of KBTO increased at the angular frequency of 10 rad/s. The storage moduli were, respectively, 2160.00 ± 73.94 Pa, 2407.50 ± 115.00 Pa, and 2560.00 ± 129.87 Pa for GO, 0.1KBGO, and 0.5KBGO hydrogels (Fig. 1g), suggesting that KBTO nanoparticles promote the cross-linking of hydrogels.

### Injectability and self-healing properties of the nanocomposite hydrogels

Since bone defect morphologies are usually irregular, it is necessary to develop an implant material to impart injectability and self-adaptive properties. We measured the viscosity of 0.1KBGO hydrogel as a function of the shear rate and discovered that the viscosity decreased with increasing shear rate, proving the injectability of 0.1KBGO hydrogel (Fig. 1h). At the same time, it was observed that the 0.1KBGO hydrogel could be continuously injected with a syringe into a glass vial filling PBS solution and remaining in gel state (insert image of Fig. 1h). Such performance allowed bone-adhesive hydrogel to be precisely injected around the fracture site through minimally invasive procedures.

Next, to assess the self-healing properties of 0.1KBGO hydrogel, strain amplitude sweep tests were performed. As depicted in Fig. 1i, as the strain increased to almost 20%, the values of G’ and G’’ remained unchanged. However, when the strain was further increased to 650%, there was a pronounced decline in G’ and a rapid ascent in G’’, culminating in their intersection—the critical point signifying the collapse of the hydrogel network. Subsequently, building upon the strain sweep test results, consecutive alternating strain sweep tests were carried out to assess the autonomous healing behavior of the 0.1KBGO hydrogel (Figure S9, Supporting Information). Initially, at a low strain (5%), the values of G’ were larger than that of G’’, indicating that the 0.1KBGO hydrogel was a stable elastic solid. Upon increasing the strain to 1000%, G’ decreased while G’’ increased, with G’ less than G’’, suggesting the collapse of the hydrogel network. Nevertheless, upon reapplication of a low strain (5%), G’ and G’’ values nearly reverted to their original levels even after five alternating cycles, which indicated the restoration of the hydrogel structure, with the mechanical properties remaining essentially unchanged both before and after the self-healing process. The rapid sol-gel state transition observed in the 0.1KBGO hydrogel underscored its remarkable self-healing capability. Macroscopic self-healing tests were also performed to demonstrate the healing behavior of hydrogels (Fig. 1j). Two prepared hydrogel disks (Φ 20 mm × 3 mm), one of which was stained red with Rhodamine B dye, were symmetrically cut into two pieces. Then, after connecting them with exchanged pieces and standing for 15 min at 37 ℃, the two pieces could completely heal with obvious cross-penetration of dye at the junction and maintaining integrity under stretch. Briefly, the Schiff base dynamic bonds formed by the amino groups on KBTO nanoparticles and the aldehyde groups on OCS endowed the 0.1KBGO hydrogel with excellent self-healing properties to reconstruct its internal structure without any external intervention. Furthermore, the self-healing effectiveness of the 0.1KBGO hydrogel was quantified through tensile testing. The stress-strain curve (Figure S10, Supporting Information) revealed a similarity between the original sample and the self-healed hydrogel, suggesting that the hydrogel has excellent self-healing ability.

### Mechano-electric response properties of the ultrasound-powered nanocomposite hydrogels

**Fig. 2:**
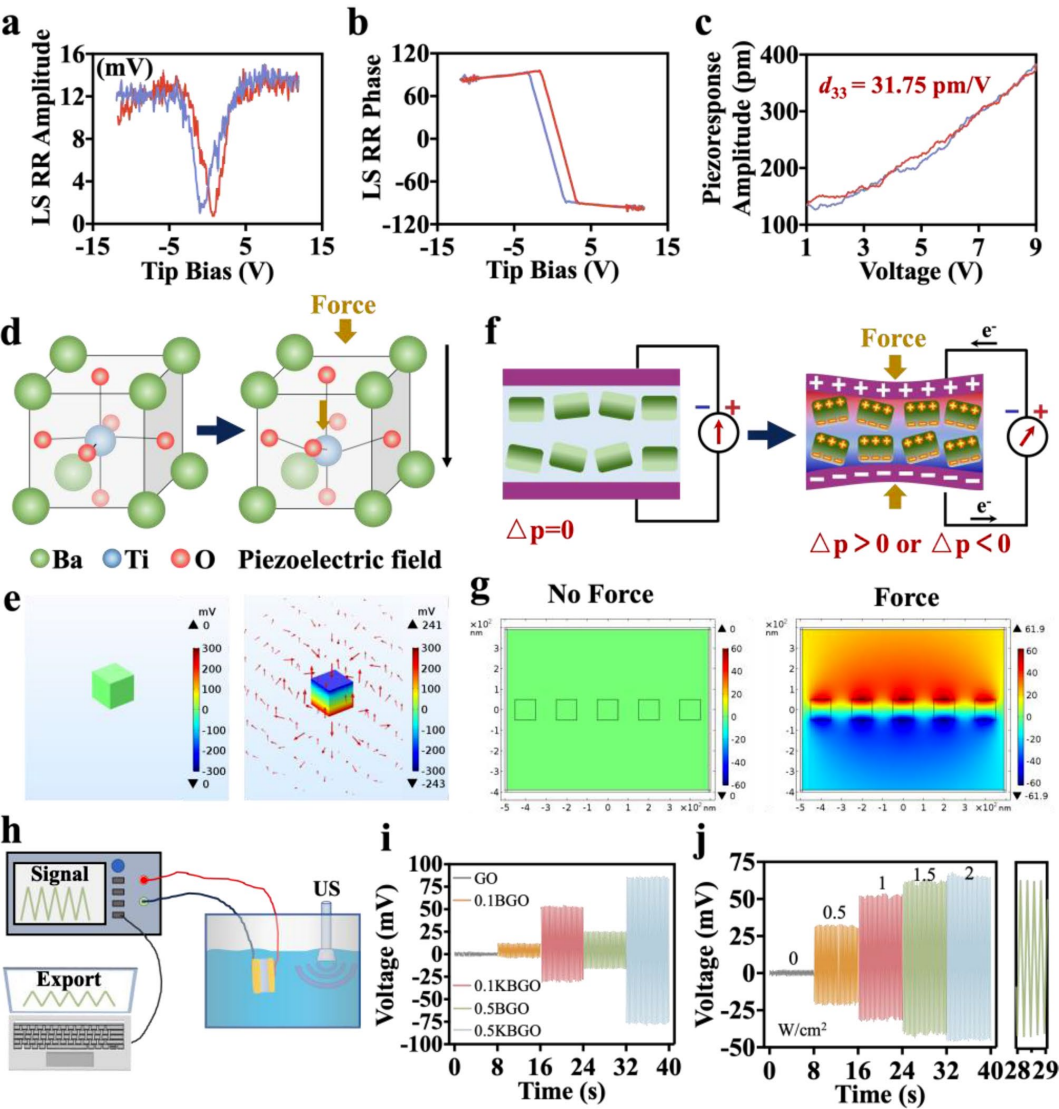
Mechano-electric response properties of the ultrasound-powered hydrogels. Piezo-response amplitude hysteresis loops (**a**) and phase curves (**b**) of KBTO nanoparticles. (**c**) Quantitative piezo-response amplitude measures the effective longitudinal piezoelectric coefficient of KBTO. (**d**) Schematic diagram of crystal structure changes of KBTO nanoparticle under mechanical force. (**e**) Finite-element simulation of the potential distribution of KBTO nanoparticle with COMSOL with or without pressure generated by ultrasound cavitation. (**f**) Schematic diagram of ultrasound-powered hydrogel generating voltage under pressure. (**g**) Finite-element simulation of the potential distribution of 0.1KBGO hydrogel without external force and under a constant 10^8^ Pa force. (**h**) Schematic diagram of a laboratory-made piezoelectric voltage outputs test apparatus. (**i**) The output voltage of GO, 0.1BGO, 0.1KBGO, 0.5BGO, and 0.5KBGO hydrogels under ultrasonic stimulation with a sound intensity of 1 W/cm^2^. (**j**) The output voltage of 0.1KBGO hydrogel under ultrasonic stimulation with different sound intensities of 0 to 2 W/cm^2^ and the enlarged view under ultrasonic stimulation with a sound intensity of 1.5 W/cm^2^

To examine the ferroelectricity of KBTO nanoparticles, atomic force microscopy (AFM) was applied. As depicted in Figure S11a, the AFM image showed the morphology of KBTO nanoparticles. With the application of an alternating bias electric field of 12 V, the amplitude of the KBTO nanoparticles changed by approximately 500 pm (Figure S11b, Supporting Information). In addition, a phase deflection of 180° was observed in the same nanoparticle (where yellow and brown represent different phase angles), indicating the ferroelectric polarization transformation process of KBTO nanoparticles (Figure S11c, Supporting Information). These above results confirmed that KBTO nanoparticles exhibit characteristic piezoelectricity and ferroelectricity. The local piezoelectric hysteresis loop and phase curve also proved the excellent ferroelectricity of KBTO. The typical butterfly hysteresis loop (Fig. 2a) indicated that the strain varies induced by the bias electric field and proved the excellent piezoelectricity response of the KBTO nanoparticles. Additionally, the local piezoelectric phase curve of KBTO nanoparticles presented a hysteresis and a 180° phase switching (Fig. 2b). The piezoelectric coefficient is a unique parameter that reflects the linear relationship between mechanical and electrical quantities of piezoelectric materials. By fitting the approximate effective piezoelectric coefficient obtained from the AFM, the piezoelectric coefficient (*d*_33_) of the KBTO nanoparticles was about 31.75 pm/V (Fig. 2c). These results validated the excellent ferroelectric responsiveness and stable ferroelectric conversion ability of KBTO nanoparticles.

According to the fundamentals of ferroelectric crystal structure, tetragonal KBTO nanoparticles are a typical non-centrosymmetric crystal (Fig. 2d) [53]. Since the titanium ions are slightly off-center in the TiO_6_ octahedron, the positive and negative ion centers are misaligned, transforming the unit cell into an effective electric dipole [54]. Under the applied mechanical force, the titanium ions further deviated from the center of the TiO_6_ octahedron and carried different charges on the surface of the KBTO nanoparticle to generate a piezoelectric potential [55, 56]. Inspired by these findings, we considered that the KBTO nanoparticles could undergo lattice deformation in response to the external pressure induced by ultrasound and form potential differences on their surfaces. Therefore, the potential distributions of KBTO nanoparticle under the pressure (10^8^ Pa) generated by ultrasound cavitation were simulated by finite-element simulation with COMSOL, which yielded a piezoelectric potential value of 241 mV for the KBTO nanoparticle (Fig. 2e).

Inspired by the excellent force-electrical conversion properties of tetragonal KBTO nanoparticles, the electrical signals of piezoelectric nanocomposite hydrogels embedded with KBTO nanoparticles were detected. The piezoelectric hydrogel was deformed under mechanical forces, resulting in increased variation in the dipole moments (ΔP) and consequent generation of electrical signals [57]. As shown in Fig. 2f, the dipoles inside the hydrogel pointed in all directions without external forces. Therefore, the macroscopic potential of KBGO piezoelectric hydrogel was maintained at 0 mV. Once an external force compressed the hydrogel, the dipoles would rotate, resulting in variations in the ΔP, forming a potential difference on the surface, generating an electrical signal in the external circuit [58]. To further validate the contribution of KBTO piezoelectric nanoparticles to the enhancement of the output voltage of the hydrogels under stress transfer, finite-element simulation with COMSOL was performed. It was found that the piezoelectric potential of piezoelectric nanocomposite hydrogel was 0 mV without external force (Fig. 2g) and 36.4 mV under lower pressure (Figure S12, Supporting Information). However, when a force of 10^8^ Pa was applied to simulate the pressure generated by ultrasonic cavitation (1.5 W/cm^2^), each KBTO nanoparticle generated a surface piezoelectric potential vertically, resulting in a total piezoelectric potential value of approximately 61.9 mV (Fig. 2g).

A laboratory-made piezoelectric voltage outputs test apparatus (Fig. 2h) was applied to detect the output voltages of hydrogels incorporated with different concentrations of KBTO (0, 0.1, and 0.5% w/v) and BTO (0.1, and 0.5% w/v) piezoelectric nanoparticles and under various ultrasound intensities (0, 0.5, 1, 1.5, and 2 W/cm^2^). Under a constant ultrasound intensity of 1 W/cm^2^ (Fig. 2i), no obvious electrical signal was detected in the GO hydrogel, indicating that ultrasound alone did not interfere with electrical signal detection. While the hydrogels incorporated with piezoelectric nanoparticles were all able to detect the electrical signal. Notably, the output voltages of KBGO hydrogels were all larger than those of BGO hydrogels. The significant increase in output voltage is pertinent to the mechanical interface between the KBTO nanoparticle surfaces and the hydrogel substrates. When no effective chemical bonds are formed between the nanoparticles and the polymer chains, the particles acting as fillers barely respond to polymer deformation [59, 60]. Interestingly, the amino-functionalized KBTO nanoparticles utilized in this study were linked with the polymer to form Schiff base bonds and hydrogen bonds that effectively concentrate the stress in the polymer chains to the piezoelectric crystal to assist the process of converting mechanical force into electrical signals. Moreover, the output voltage of 0.1KBGO hydrogel under various ultrasound intensities was further investigated. As depicted in Fig. 2j, the output electrical signals generated by 0.1KBGO hydrogel increased with enhanced ultrasound intensity. The output voltage generated by 0.1KBGO hydrogel under ultrasound intensities of 1.5 W/cm^2^ (-41.16 to 61.82 mV) and 2 W/cm^2^ (-45.19 to 65.68 mV) were both within the range of electrical stimulation intensity for piezoelectric bone healing [61, 62]. The above results ensured that the piezoelectric hydrogel could generate continuous electrical stimulation to bone tissue under ultrasound vibration, which is widely thought to have huge potential applications to accelerate bone healing. However, to reduce the influence, ultrasound with an intensity of 1.5 W/cm^2^ was used in subsequent in vivo and in vitro experiments.

### In vitro adhesion properties of the ultrasound-powered nanocomposite hydrogels

**Fig. 3:**
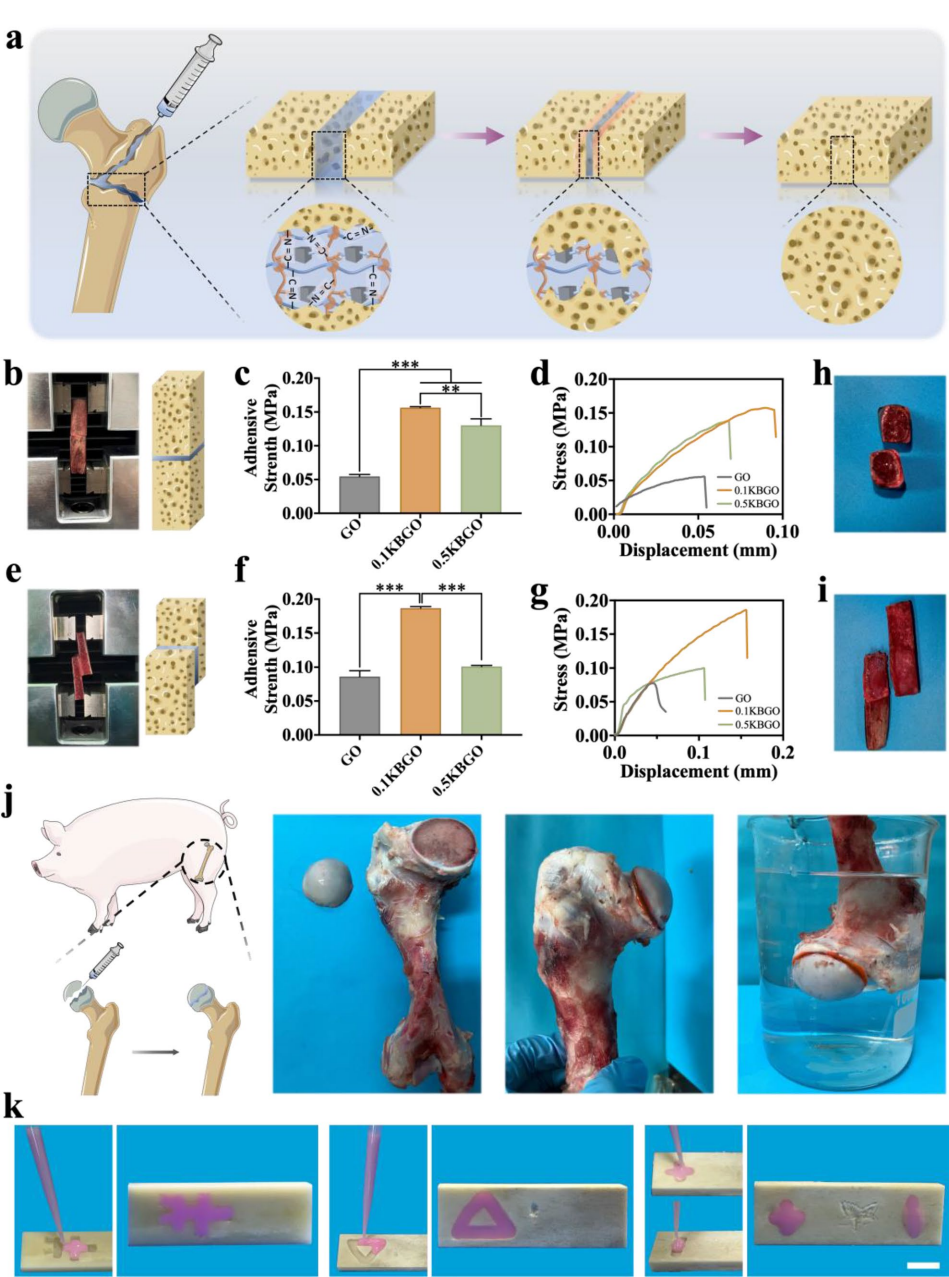
Adhesion properties of ultrasound-powered hydrogels. (**a**) Schematic illustration of the KGBO piezoelectric hydrogel fixing bone fragments and facilitating bone regeneration. (**b**) The experiment of end-to-end adhesion strength test. (**c**) The end-to-end adhesion strength of GO, 0.1KBGO, and 0.5KBGO hydrogels. (**d**) Stress-displacement curves of end-to-end adhesion tensile test. (**e**) The experiment of lap-shear adhesion strength test. (**f**) The lap-shear adhesion strength of GO, 0.1KBGO, and 0.5KBGO hydrogels. (**g**) Stress-displacement curves of lap-shear adhesion tensile test. (**h**) Adhesion interfaces of end-to-end adhesion. (**i**) Adhesion interfaces of lap-shear adhesion. (**j**) Images of glued pig femur bone pieces with 0.1KBGO piezoelectric hydrogel. (**k**) The ultrasound-powered self-adaptive hydrogel filling irregularly shaped bovine bone defects. Scale bar represents 2 cm. ANOVA followed by Tukey’s post hoc test was performed for statistical analysis (*p < 0.05, **p < 0.01, ***p < 0.001)

Implant materials used for complex bone defect healing frequently need to be bone-adhesive to avoid inflammation and damage to surrounding tissues brought on by material movement. Herein, the ultrasound-powered nanocomposite hydrogel and bone tissue were bonded together through carbon-nitrogen double bonds to create an adhesive surface (Fig. 3a). To investigate the adhesion ability of GO, 0.1KBGO, and 0.5KBGO hydrogels, we adhered the porcine cancellous bone fragments in both end-to-end and lap-shear manners for subsequently dynamic tensile tests. When the end-to-end manner was adopted (Fig. 3b-d), the GO hydrogel without KBTO nanoparticles exhibited the lowest adhesive strength. However, embedding KBTO could significantly improve the adhesive strength of the nanocomposite hydrogels. Interestingly, the adhesive strength of 0.1KBGO was 0.156 ± 0.001 MPa, which was significantly higher than that of 0.5KBGO (0.130 ± 0.010 MPa) and GO hydrogel (0.055 ± 0.003 MPa). It is generally reported that adhesive strength is related to material cohesion and interfacial adhesion [49]. On the one hand, the embedded KBTO nanoparticles increase the cross-linking density and thus enhance the cohesion of the 0.1KBTO hydrogel. On the other hand, the hydrogen bonds formed by the amino groups on KBTO nanoparticles and the hydroxyl groups on the bone tissues also contribute to enhanced interfacial adhesion. It is worth noting that the adhesive strength decreased as the concentration of KBTO nanoparticles increased up to 0.5% w/v, which may be attributed to the fact that extra KBTO nanoparticles consumed aldehyde groups, thereby reducing the interfacial adhesion of hydrogel. Similarly, the above trends were also observed when the lap-shear manner was adopted (Fig. 3e-g). The maximum adhesive strength of 0.1KBGO was 0.19 ± 0.02 MPa, which was 2.17 and 1.85-fold higher than GO and 0.5KBGO, respectively. In addition, a cohesive failure pattern exited on cancellous bone fragments adhered by 0.1KBGO hydrogel in both end-to-end (Fig. 3h) and lap-shear (Fig. 3i) manners, and the hydrogel-tissue interfaces were well preserved, which demonstrated the excellent interfacial adhesion of the 0.1KBGO hydrogel. After quantifying the adhesive strength, we attempted to adhere freshly sawn bone fragments onto the porcine femoral section in vitro by injecting 0.1KBGO hydrogel and immersed the adhesive surface in PBS solution at 37 °C to simulate the in vivo environment (Fig. 3j). As expected, the adhesive surface exhibited good stability with satisfactory adhesion properties, which indicated that the ultrasound-powered nanocomposite hydrogel exhibited high-efficiency bone adhesion properties and was anticipated to treat irregular bone defects. Moreover, the presence of the aldehyde groups on OCS and the amino groups on Gel and KBTO enabled this ultrasound-powered hydrogel not only to have satisfactory bone-adhesive properties, but also to exhibit a self-adaptive filling irregularly shaped bone defects in bovine bone (Fig. 3k).

### Ultrasound-powered bone-adhesive hydrogels have satisfactory biocompatibility in vitro

To construct a bone-mimicking mechano-electric response microenvironment, the in vitro biocompatibility of ultrasound-powered hydrogels incorporating KBTO nanoparticles was evaluated. According to the osteogenesis-friendly electrical stimulation generated by the KBGO hydrogel at different ultrasound intensities, the following ultrasound parameters were used: 2.0 MHz, 1.5 W/cm^2^, 50% duty cycle, and 5 min. After incubating bone mesenchymal stem cells (BMSCs) on the surfaces of hydrogels for 48 h, a Live/Dead staining assay was performed. Only a few dead cells (red fluorescence) were observed in each group, indicating that these piezoelectric hydrogels were not cytotoxic (Figure S13a, Supporting Information). To obtain a KBGO hydrogel with an optimal biological effect, the concentration of KBTO nanoparticles was optimized according to the cell proliferation results evaluated by the cell counting kit 8 (CCK-8) assay. The results (Figure S13b, Supporting Information) showed that cells in all samples exhibited good proliferation during culturing. The optical density (OD) value of the GO + US group was similar to the GO group, with no significant difference, indicating that ultrasound stimulation alone did not affect cell proliferation. From day 4, the cell viability of BMSCs in the 0.1KBGO + US group exhibited a significant increase compared with the other five groups. Moreover, the OD value of the 0.1KBGO + US was 1.35 and 1.37-fold higher than the GO group on days 4 and 7, respectively. These results revealed that the local electric field generated by 0.1KBGO hydrogel under ultrasonic stimulation with a sound intensity of 1.5 W/cm^2^ could facilitate BMSCs proliferation. Furthermore, CLSM images of the cytoskeleton (Figure S14a, Supporting Information) displayed that the BMSCs were spindle-shaped on the surface of hydrogels in the GO and GO + US groups and were elongated polygonal stretch with filopodia protruding in other groups, especially in the 0.1KBGO + US group. According to the cell spread area quantified by ImageJ software (Figure S14b, Supporting Information), the spread area of BMSCs in the 0.1KBGO + US group was the largest, and there was a significant difference compared with the GO and GO + US groups, consistent with the above experimental results. Overall, these experiments demonstrated that electrical stimulation generated by 0.1KBGO + US could induce BMSCs proliferation and spreading, essential for promoting bone repair.

Hemocompatibility assessment is an essential part of the biosafety evaluation of implanted biomaterials. Based on the findings of the hemolysis test (Figure S15, Supporting Information), three groups showed high hemocompatibility, with hemolysis rates below 1%.

### The electrical stimulation generated by ultrasound-powered nanocomposite hydrogel facilitates the osteogenic differentiation of BMSCs

**Fig. 4:**
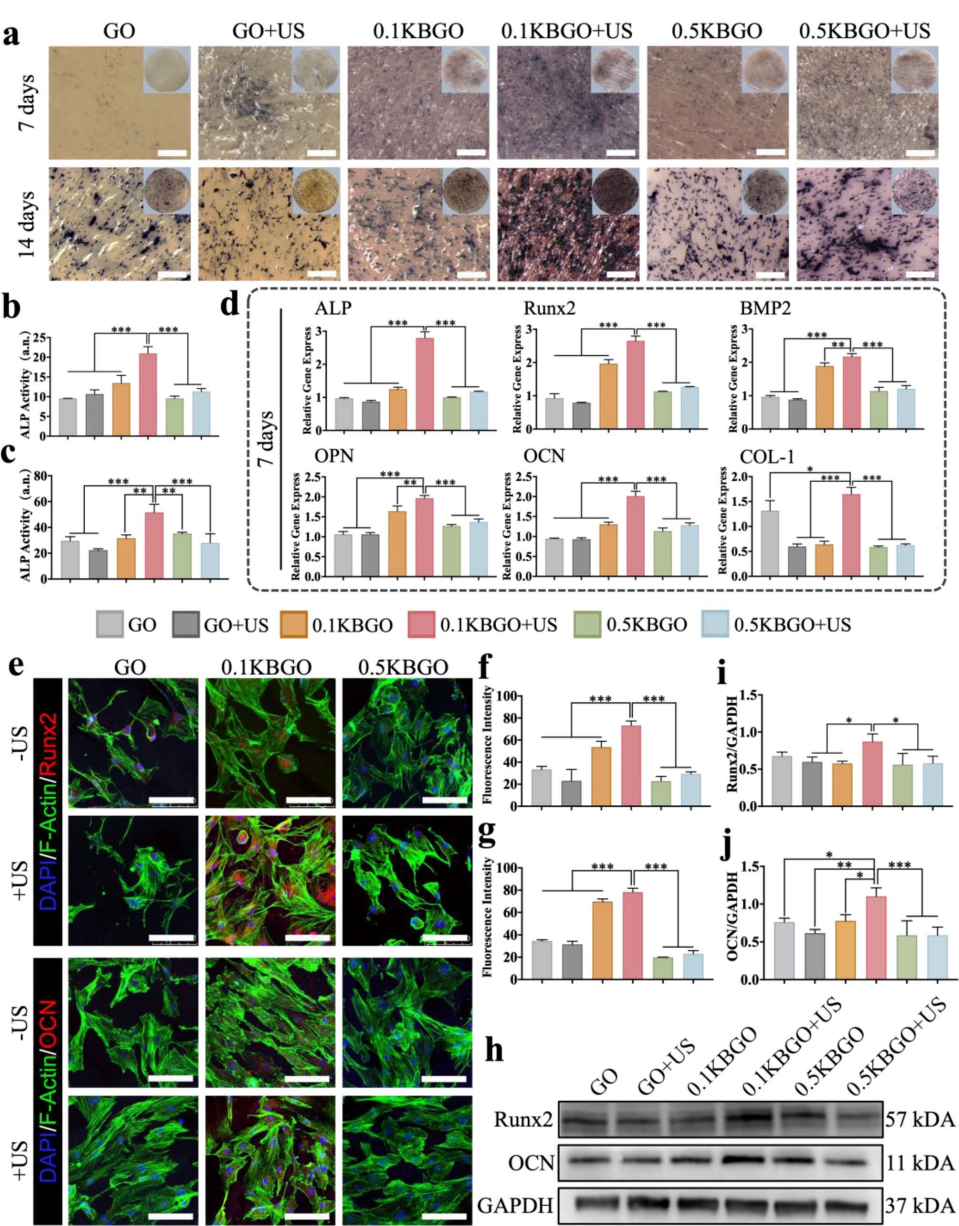
In vitro osteogenic differentiation of BMSCs on ultrasound-powered hydrogel surfaces. (**a**) ALP staining of BMSCs incubated on each hydrogel surface for 7 and 14 days. Scale bar represents 500 μm. (**b**, **c**) Quantitative analysis of ALP staining for 7 and 14 days (*n* = 3). (**d**) The expression levels of osteogenic genes (ALP, Runx2, BMP2, OPN, OCN, and COL-I) of BMSCs cultured on different samples on day 7 (*n* = 3). (**e**) Immunofluorescent staining of osteogenesis-related protein Runx2 and OCN (red), cytoskeleton (green), and nuclei (blue) of BMSCs cultured on each hydrogel for 7 days. Scale bar represents 100 μm. (**f**, **g**) Quantitative analysis of fluorescence intensity of Runx2 and OCN (*n* = 3). (**h**) Western blot assay of Runx2 and OCN of BMSCs on day 7. (**i**, **j**) Quantitative analysis of protein band intensities (*n* = 3). ANOVA followed by Tukey’s post hoc test was performed for statistical analysis (*p < 0.05, **p < 0.01, ***p < 0.001)

To investigate the in vitro osteogenic ability of the electrical stimulation generated by ultrasound-powered nanocomposite hydrogels, BMSCs were seeded and co-cultured on different samples. Qualitative images of alkaline phosphatase (ALP) staining on days 7 and 14 displayed that ALP activity significantly increased in the 0.1KBGO + US group compared with other groups (Fig. 4a). As illustrated in the ALP quantitative assay on days 7 and 14(Fig. 4b, c), no significant difference was detected between the GO and GO + US groups besides the effect of ultrasound stimulation alone on osteogenic differentiation of BMSCs. The 0.1KBGO + US group exhibited the highest ALP activities (20.97 ± 1.71 a.n. and 51.69 ± 6.18 a.n.), which were almost 2.19 and 1.74-fold higher than that of the GO group, 1.56 and 1.63-fold higher than that of the 0.1KBGO group on the days 7 and 14. ALP is widely recognized as a marker of early osteogenesis differentiation. These results demonstrated that the mechano-electric response microenvironment generated by 0.1KBGO hydrogel under ultrasonic stimulation could induce rapid and effective osteogenic differentiation of BMSCs. In addition, RT-qPCR was carried out on days 7 (Fig. 4d) and 14 (Figure S16, Supporting Information) to assess the expression level of osteogenic-related genes. The results suggested that ultrasound alone had no effect on the expression of osteogenic-related genes. Compared to the GO group, the 0.1KBGO group had greater levels of osteogenic-related gene expression except collagen I (COL-I). Combined with the previous rheological analysis, it is speculated that it is related to the enhancement of the mechanical properties of the hydrogel [63]. However, is was more noteworthy that the 0.1KBGO + US group exhibited the highest expression than other groups, including both early, intermediate, and late osteogenic genes namely ALP, runt-related transcription factor 2 (Runx2), bone morphogenetic protein 2 (BMP2), osteopontin (OPN), osteocalcin (OCN), and COL-I, further corroborating that 0.1KBGO hydrogel with ultrasonic triggering provides a favorable electrophysiological environment for osteogenesis.

Generally, alterations at the gene level are paralleled by changes at the protein level, but this relationship may be subject to the influence of factors such as modification and splicing. Accordingly, further investigations of osteogenesis-related proteins are essential. Consistently, the immunofluorescence staining assay showed the highest expression levels of early and late osteogenic-related proteins Runx2 and OCN (red fluorescence) in the 0.1KBGO + US group (Fig. 4e). The corresponding semi-quantitative analysis (Fig. 4f and g) confirmed that compared with the GO group, the fluorescence intensity of Runx2 and OCN in the 0.1KBGO + US group increased by 120.31% and 126.88%, respectively. As expected, western blot results depicted that the protein expressions of Runx2 and OCN were the highest in the 0.1KBGO + US group compared to the other five groups (Fig. 4h-j). Herein, we have proposed and evidenced an ultrasound-powered nanocomposite hydrogel based on non-centrosymmetric KBTO nanoparticles. Notably, KBTO could respond to the ultrasonic-induced microscopic pressure to establish a built-in electric field, subsequently providing a microenvironment favorable for osteogenesis. Overall, our results substantiated that the electrical stimulation produced by 0.1KBGO hydrogel in response to ultrasound provided an optimal extracellular mechano-electrical physiological environment to manipulate the osteogenic differentiation of BMSCs.

### The electrical stimulation generated by ultrasound-powered bone-adhesive nanocomposite hydrogel accelerates bone healing

**Fig. 5:**
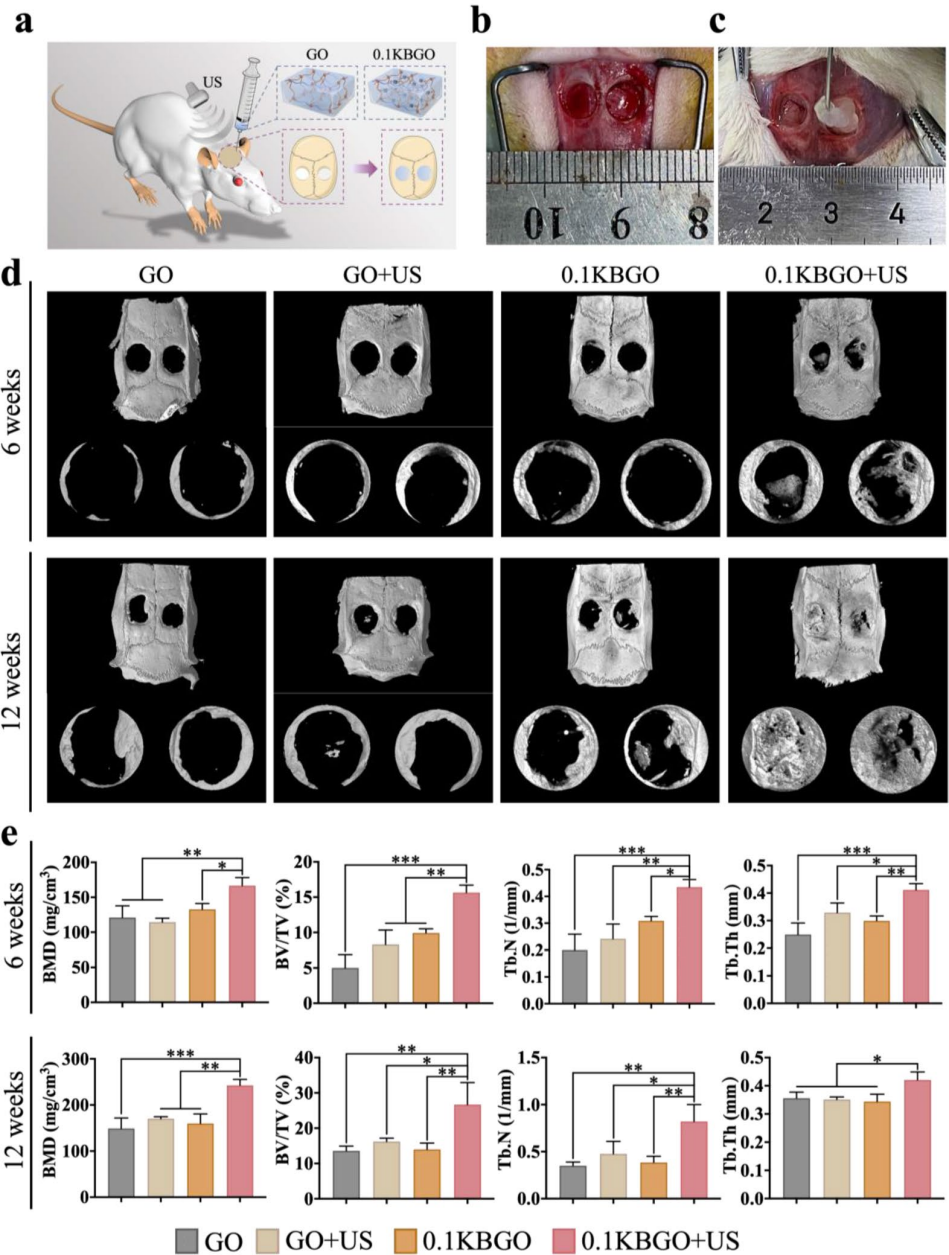
In vivo new bone regeneration of rat critical-size calvarial defects in different groups. (**a**) Schematic illustration of the rat critical-size calvarial defects (d = 5 mm) surgical procedure and different treatments. (**b**, **c**) Optical photos of the implantation of the injectable GO and 0.1KBGO hydrogels into the 5 mm rat calvarial defects. (**d**) Micro-CT images showing cross-sectional bone regeneration in different groups at weeks 6 and 12 postoperatively. (**e**) Quantitative analysis of bone regeneration within ROI at weeks 6 and 12 (*n* = 3). ANOVA followed by Tukey’s post hoc test was performed for statistical analysis (*p < 0.05, **p < 0.01, ***p < 0.001)

Inspired by the significant in vitro osteogenic advantage of electrical stimulation generated by 0.1KBGO + US group, we selected GO and 0.1KBGO hydrogels to conduct a rat critical-size calvarial bone defect model (d = 5 mm) according to the surgical procedure illustrated in Fig. 5a to further study new bone formation in vivo. Before implanting hydrogels, we first assessed the in vivo toxicity reaction. No pathological changes were observed, demonstrating that these hydrogels have great biocompatibility in vivo (Figure S17, Supporting Information). Figure 5b and c displayed the surgical photographs of the implantation site in the rat calvarial defect. After 6 and 12 weeks, Micro-CT scanning and histological analysis of the harvested calvarial tissues were performed. As shown in Fig. 5d and Figure S18 (Supporting Information), newly formed bone tissue was visualized in all groups, while the most newly bone tissue was observed in the 0.1KBGO + US group. Various bone parameters such as bone mineral density (BMD), bone volume/total volume (BV/TV), trabecular number (Tb.N), and trabecular thickness (Tb.Th) were measured in Fig. 5e. The highest values for these bone parameters were observed in the 0.1KBGO + US group, followed by the 0.1KBGO group and the GO or GO + US group.

**Fig. 6:**
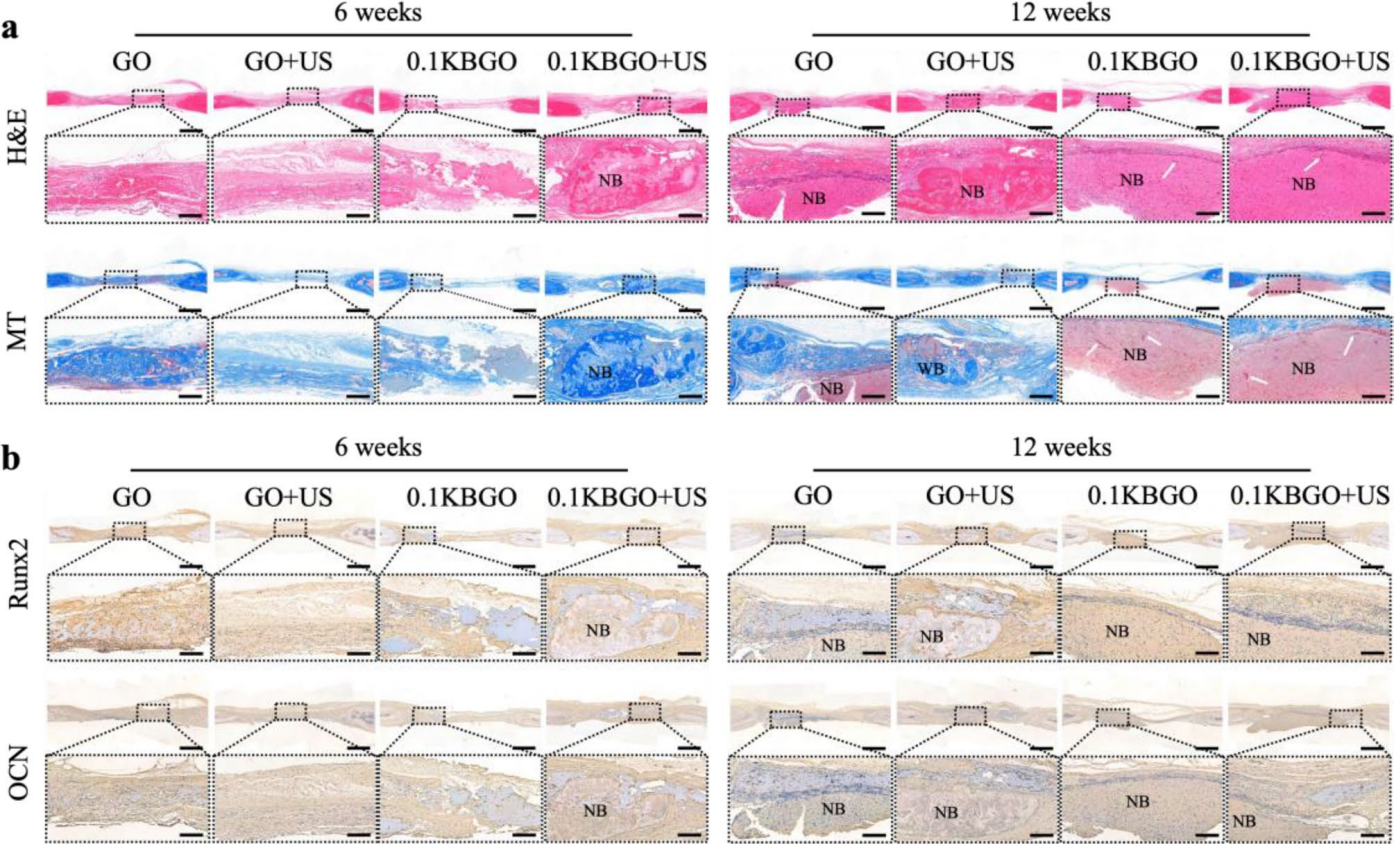
Histological analysis of newly formed bone tissue at 6 weeks and 12 weeks after surgery. (**a**) H&E and MT staining of demineralized calvarial sections. Higher magnification images are taken from the areas enclosed by a square in the upper row. (**b**) Runx2 and OCN immunohistochemical staining of demineralized calvarial sections. Higher magnification images are taken from the areas enclosed by a square in the upper row. WB: woven bone, NB: newly formed bone. The white arrow represents newly formed bone marrow. Scale bars represent 1 mm (lower magnification) and 200 μm (higher magnification)

Moreover, hematoxylin & eosin (H&E) and Masson’s trichome (MT) staining were conducted in the tissues harvested from the calvarial defect and the adjacent regions. 6 weeks after implantation, H&E staining (Fig. 6a) showed that the center of the bone defect was mainly filled with connective tissue in the GO and GO + US groups, with a small amount of new bone tissue at the edge of the defect area in the 0.1KBGO group. Fortunately, osteoid tissue and a trip of woven bone (red staining) were observed in the 0.1KBGO + US group. Newly formed bone tissue appeared mainly as blue-stained areas, with a few red-stained areas in MT staining images (Fig. 6a). On week 12, a large amount of newly formed bone tissue was observed in the GO, GO + US, and 0.1KBGO groups. As expected, the most regenerated bone tissue was observed throughout the defect region in the 0.1KBGO + US group, nearly closing the defect, consistent with the Micro-CT results. Interestingly, the regenerated bone tissue appeared as mature lamellar bone with new bone marrow formation (white arrows), staining red in H&E and MT staining images.

It is documented that BMSCs can be recruited to bone defect areas and differentiate into osteoblasts contributing to bone regeneration [64]. Consequently, the recruitment and differentiation of host BMSCs are pivotal for bone repair after implantation. Next, we conducted immunohistochemistry staining to assess the expression levels of two osteogenic differentiation markers, Runx2 and OCN. The immunohistochemistry staining (Fig. 6b) revealed that more Runx2 and OCN positive cells were detected in the 0.1KBGO + US group than in the other groups, which evidenced that the local electric field produced by 0.1KBGO hydrogel implanted in the bone defect region in response to ultrasonic vibration could facilitate the osteogenic differentiation of BMSCs and provide a favorable environment for bone regeneration.

In addition, we investigated the biodegradability of piezoelectric hydrogels in vivo. Biomaterials that degrade continuously with tissue regeneration not only provide a sufficient space for the tissue to repair, but also eliminate the need for secondary surgery to remove the material from the injury site [65]. The biodegradability of hydrogels was assayed by implanting these materials subcutaneously in rats. As shown in Figure S19a (Supporting Information), 2 weeks after implantation, the GO hydrogel was mostly degraded by an enzymatic reaction. However, the presence of KBTO nanoparticles accounted for the significantly lower degradation rate of 0.1KBGO compared to GO. After 6 weeks, the diameter and thickness of GO and 0.1KBGO hydrogels both significantly decreased, and their margins with the tissue were blurred. Given that the regeneration and remodeling of bone defects usually take months to years, the degradation rate of 0.1KBGO hydrogel can match the regeneration rate of bone defects. Moreover, a small number of inflammatory cells infiltrating the implantation area was visible in the H&E staining images (Figure S19b, Supporting Information), but no obvious inflammatory reaction was visible, indicating that these hydrogels had satisfactory histocompatibility.

### Ultrasound-powered hydrogels stimulate the osteogenic differentiation of BMSCs through ERK/MAPK and PI3K-AKT signaling pathways

**Fig. 7:**
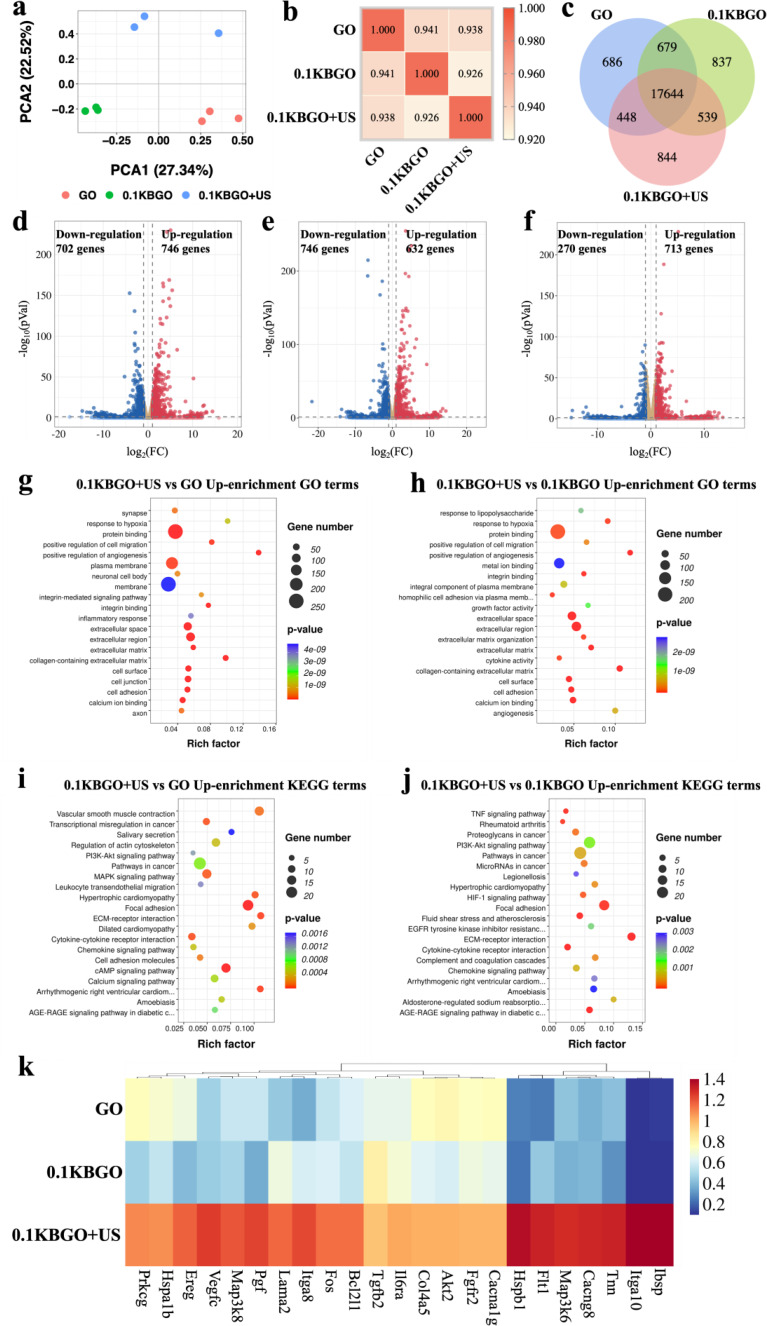
Transcriptome analysis of BMSCs gene expression in different groups. (**a**) PCA analysis of all data of GO, 0.1KBGO, and 0.1KBGO + US groups. (**b**) Pearson correlation coefficient heatmap of all samples. (**c**) Venn diagram of the number of differentially expressed genes among GO, 0.1KBGO, and 0.1KBGO + US groups. (**d**-**f**) Volcano analysis of differentially expressed genes of 0.1KBGO + US vs. GO, 0.1KBGO + US vs. 0.1KBGO, and 0.1KBGO vs. GO. (**g**-**h**) The top 20 significant up-enriched GO terms of 0.1KBGO + US vs. GO and 0.1KBGO + US vs. 0.1KBGO. (**i**-**j**) The top 20 significant up-enriched KEGG pathways of 0.1KBGO + US vs. GO and 0.1KBGO + US vs. 0.1KBGO. (**k**) Heatmap of the represented up-regulated genes in GO, 0.1KBGO, and 0.1KBGO + US. *n* = 3 biological replicates per group

To further understand the mechanism of BMSCs osteogenic differentiation facilitated by electrical stimulation, RNA sequencing was conducted in the GO, 0.1KBGO, and 0.1KBGO + US groups to analyze the gene expression profiles. First, principal component analysis (PCA) (Fig. 7a) of the samples in three groups was performed using the function princomp. The results demonstrated that the transcriptome data could be utilized for further analysis. Then the inter-sample repeatability was evaluated by the Pearson correlation coefficient, which helped to eliminate outlier samples. As shown in Fig. 7b, most correlation coefficients were within acceptable limits (R^2^ > 0.90). A Venn diagram (Fig. 7c) of all differentially expressed genes (DEGs) showed many differences. 686 genes were GO-specific genes, 837 genes were 0.1KBGO-specific genes, and 844 genes were 0.1KBGO + US-specific genes. Our results indicated that the promoted osteogenic differentiation of BMSCs was piezoelectric field dependent. In addition, the overlap of 17,644 genes among the GO, 0.1KBGO, and 0.1KBGO + US groups might be related to implanted scaffolds. The following volcano plots (Fig. 7d and f) also revealed a wide range of gene expression differences, including 746 up-regulated and 702 down-regulated genes (0.1KBGO + US versus GO), 632 up-regulated and 746 down-regulated genes (0.1KBGO + US versus 0.1KBGO), and 713 up-regulated and 270 down-regulated genes (0.1KBGO versus GO). Furthermore, the gene ontology (GO) database analysis was performed to evaluate the collected up-regulated differentially expressed genes in 0.1KBGO vs. GO, 0.1KBGO + US vs. GO, and 0.1KBGO + US vs. 0.1KBGO in three categories: biological processes (BP), molecular function (MF), and cellular components (CC). As exhibited in Fig. 7g and h, the up-regulated genes in the 0.1KBGO + US group were mainly enriched in protein binding, integrin binding, cell adhesion, and calcium ion binding. In contrast, in the 0.1KBGO group, the up-regulated genes were present in immune response, defense response, and chemokine-related pathways (Figure S20, Supporting Information). Furthermore, the Kyoto Encyclopedia of Genes and Genomes (KEGG) pathway enrichment analysis was also performed to reveal the function of DEGs and to analyze potential signaling pathways. The top 20 up-regulated KEGG pathways in the 0.1KBGO + US group was shown in Fig. 7i and j. In addition to focal adhesion and cell adhesion molecules, phosphatidylinositol 3-kinase (PI3K)/ protein kinase B (AKT) signaling pathway, mitogen-activated protein kinase (MAPK) signaling pathway, and calcium signaling pathway were also up-regulated. Nevertheless, the pathways related to calcium transport and osteogenesis were not enriched in the 0.1KBGO group (Figure S21, Supporting Information). Therefore, we next focused on the differences in osteogenesis-related gene expressions induced by the electrical signal generated by piezoelectric hydrogel during ultrasound cavitation, including the PI3K/AKT, MAPK, and calcium signaling pathways. Subsequently, these DEGs were displayed in a heatmap (Fig. 7k).

**Fig. 8:**
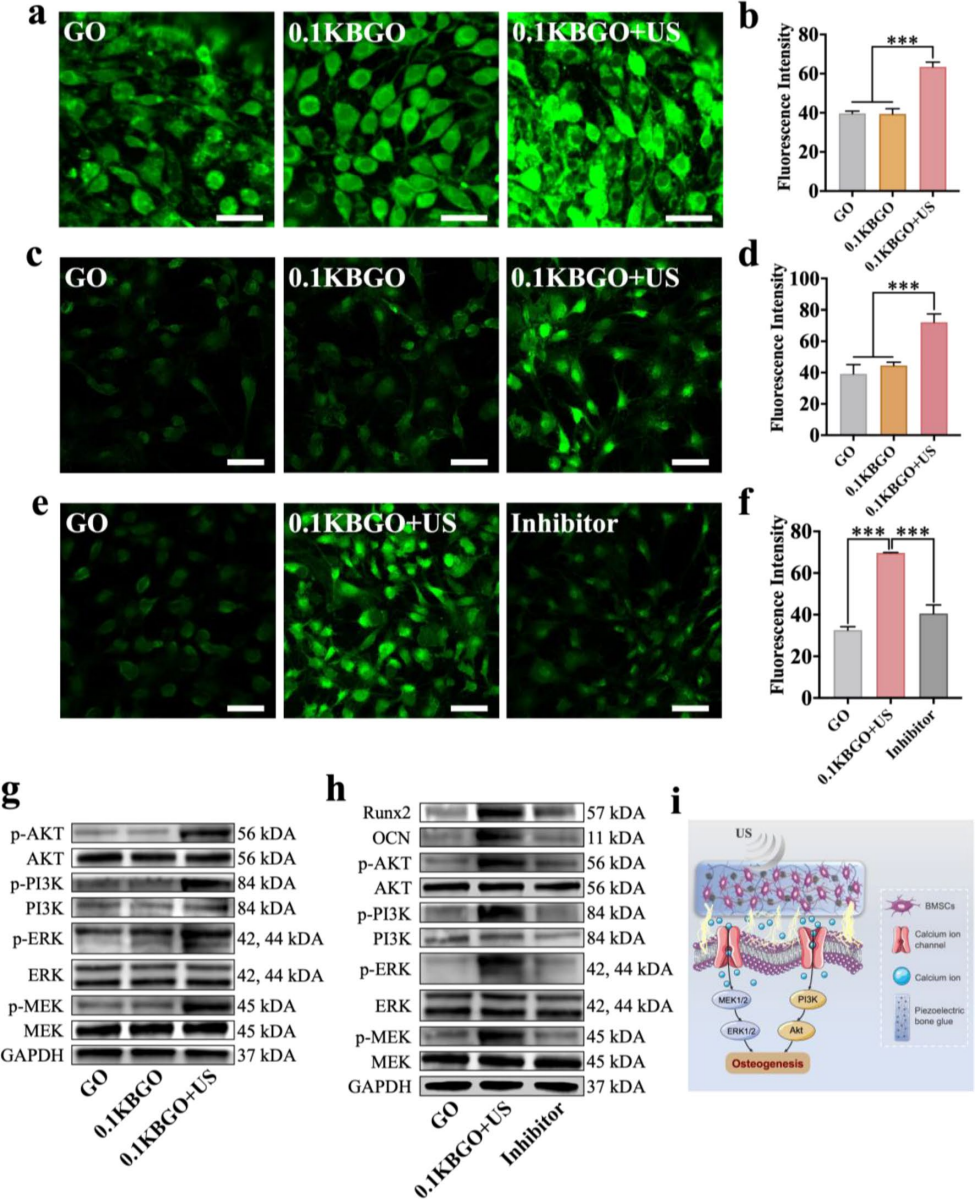
Ultrasound-powered hydrogel facilitates BMSCs osteogenic differentiation by increasing Ca^2+^ influx and active PI3K/AKT and MEK/ERK pathways. (**a**) Detections of Vm in different groups. Scale bar represents 50 μm. (**b**) Quantitative analysis of immunofluorescence intensity of Vm (*n* = 3). (**c**) Immunofluorescent staining of intracellular Ca^2+^ (green) of BMSCs in GO, 0.1KBGO, and 0.1KBGO + US groups. Scale bar represents 100 μm. (**d**) Quantitative analysis of immunofluorescence intensity of intracellular Ca^2+^ (*n* = 3). (**e**) Immunofluorescent staining of intracellular Ca^2+^ (green) of BMSCs in GO, 0.1KBGO, and 0.1KBGO + US groups after Ca^2+^ influx inhibited by GdCl_3_. Scale bar represents 100 μm. (**f**) Quantitative analysis of immunofluorescence intensity of intracellular Ca^2+^ after Ca^2+^ influx inhibited by GdCl_3_ (*n* = 3). (**g**) Western blot assay of the phosphorylation levels of PI3K, AKT, MEK, and ERK in BMSCs in different groups. (**h**) Western blot assay of the phosphorylation levels of PI3K, AKT, MEK, and ERK and osteogenesis-related proteins Runx2 and OCN in BMSCs after Ca^2+^ influx inhibited by GdCl_3_. (**i**) Schematic illustration of the molecular mechanism of the ultrasound-powered hydrogel facilitating osteogenesis. ANOVA followed by Tukey’s post hoc test was performed for statistical analysis (*p < 0.05, **p < 0.01, ***p < 0.001)

Bioelectricity objectively exists in the living organisms [23, 66]. At the cellular level, nearly all mammalian cells possess a long-term and steady-state transmembrane potential, named membrane potential (Vm), which plays a significant role as a cell-autonomous bioelectric regulator in the control of various cellular processes such as cell cycle and proliferation [22, 67]. To investigate the changes in Vm, we observed the fluorescence intensity of each group under CLSM after staining with a DiBAC4(3) fluorescent probe. From the captured images (Fig. 8a) and the quantitative analysis of fluorescence intensity (Fig. 8b), it could be seen that the 0.1KBGO + US group had the highest fluorescence intensity, which was an indication of cell membrane depolarization. Calcium ions (Ca^2+^) are regarded as a crucial second messenger for intracellular signaling transduction. An increasing body of evidence highlights the essential role of the calcium signaling pathway in osteogenic differentiation [68, 69]. As a consequence of piezoelectric stimulation, cell membrane depolarization leads to the opening of calcium channels to increase Ca^2+^ influx [25]. To determine whether electrical stimulation of 0.1KBGO hydrogel driven by ultrasound cavitation may enhance the concentration of intracellular Ca^2+^ through calcium channels, fluo-4 acetoxymethyl ester (Fluo-4 AM) was applied to investigate the changes of intracellular Ca^2+^. The immunofluorescence photographs of intracellular Ca^2+^ in different groups and the corresponding analysis of fluorescence intensity were displayed in Fig. 8c and d, respectively. According to the results, the fluorescence intensity of the 0.1KBGO + US group was increased significantly, almost 1.84 and 1.63-fold higher than that of the GO and 0.1KBGO groups, respectively. However, no significant difference was detected between the latter two groups mentioned above. These experimental results suggested that the 0.1KBGO piezoelectric hydrogel exited alone hardly increased Ca^2+^ influx, and the electrical stimulation of piezoelectric hydrogel driven by ultrasound cavitation was essential. To further support the participation of calcium channels in this mechanism, we observed the intracellular Ca^2+^ levels after the calcium channels were inhibited by GdCl_3_. As expected, the inhibitor group with GdCl_3_ experienced a reduction in Ca^2+^ concentration to about 58.22% of the 0.1KBGO + US group (Fig. 8e and f), which indicated that electrical stimulation driven by ultrasound cavitation could increase intracellular Ca^2+^ concentration by activating calcium channels.

The increased Ca^2+^ concentration in the cytoplasm can activate phospholipase C (PLC), thereby leading to the release of Ca^2+^ from the endoplasmic reticulum, [70] resulting in a further increase in the concentration of intracellular Ca^2+^. Consequently, the downstream molecules in PI3K/AKT signaling pathway and mitogen-activated protein kinase (MEK)/extracellular regulated protein kinase (ERK) signaling pathway are triggered to activate by Ca^2+^ stimulation [71]. The PI3K/AKT and MEK/ERK pathways have been documented to be involved in a variety of signaling transduction processes, including cell proliferation, apoptosis, and differentiation [44, 72]. Especially, the PI3K/AKT pathway is also crucial for bone metabolism, which can prevent osteoporosis by facilitating osteogenic differentiation and regulating bone regeneration [73]. Consistent with the transcriptome sequencing results, the Western blot assay (Fig. 8g) of the phosphorylation levels of proteins in the PI3K/AKT and MEK/ERK pathways demonstrated that the 0.1KBGO + US group exhibited substantially higher protein expression levels of p-PI3K, p-AKT, p-MEK, and p-ERK than the other two groups, but not for PI3K, AKT, MEK, and ERK. Current evidence suggests that activating the PI3K/AKT signaling pathway improves the DNA binding of Runx2 and Runx2-dependent transcription in BMSCs [74]. In particular, Runx2 stability and transcriptional activity are increased by ERK signaling [75]. As a key transcription factor in the nucleus, Runx2 regulates the expression of osteogenic marker genes including ALP, OPN, and OCN, and the phenotype and function of osteoblasts [76]. Accordingly, it is considered a marker of early osteogenesis. OCN is a protein abundant in bone tissue and is characteristically expressed in mature osteoblasts. Similarly, we investigated the expression levels of the signaling proteins in the two pathways and osteogenic biomarkers (Runx2 and OCN) after blocking calcium channels with gadolinium trichloride (GdCl_3_), to further examine the mechanism of the mechano-electric response microenvironment of 0.1KBGO hydrogel to facilitate osteogenic differentiation of BMSCs. In the western blot analysis (Fig. 8h), it could be observed that the PI3K/AKT and MEK/ERK signaling pathways were suppressed by the inhibitor, and the effect of 0.1KBGO piezoelectric hydrogel on promoting osteogenic differentiation of BMSCs was significantly diminished.

Based on the above experimental results, we propose that electrical stimulation of piezoelectric hydrogel during ultrasound cavitation can lead to cell membrane depolarization, which is conducive to Ca^2+^ influx through the opening calcium channels. As a second messenger, Ca^2+^ can activate the PI3K/AKT and MEK/ERK signaling pathways. Consequently, the expression of downstream gene Runx2 was up-regulated, which was implicated in the regulation of subsequent osteogenesis-related genes to facilitate the osteogenic differentiation of BMSCs (Fig. 8i).

## Conclusion

Herein, we constructed an injectable ultrasound-powered bone-adhesive nanocomposite hydrogel that not only possesses the injectabilities, self-adaptive ability, and bone-adhesion required to adapt irregular defect morphologies, but also generates electrical stimulation in response to ultrasound cavitation for bone regeneration. The dynamic bonding between the amino-functionalized KBTO piezoelectric nanoparticles and the OCS/Gel network endowed the hydrogel with injectability, self-adaptability and mechano-electric responsiveness. In addition, the incorporation of KBTO nanoparticles enhanced the interfacial adhesion strength of KBGO to the bone, allowing it to form a strong adhesion with isolated porcine bone fragments. Under the appropriate pressure generated by ultrasonic cavitation, 0.1KBGO hydrogel generated a stable and bone regeneration-friendly output voltage of -41.16 to 61.82 mV. Both in vitro cell experiments and in vivo animal investigations substantiated that the electrical stimulation generated by 0.1KBGO ultrasound-powered hydrogel could promote the osteogenic differentiation of BMSCs and accelerate bone healing. Notably, we further investigated the mechanism of the electrical stimulation generated by ultrasound-powered hydrogels on osteogenic differentiation through transcriptome analysis. It was found that the process was associated with the opening of calcium ion channels activated by electrical stimulation and downstream PI3K/AKT and MEK/ERK signaling pathways, which led to the upregulation of the Runx2 gene and subsequent transcription of various osteogenic-related genes, thereby controlling the osteogenic differentiation fate of BMSCs. In conclusion, the present study provides an innovative strategy for the clinical development of novel ultrasound-powered hydrogels to fix fractured bone fragments and accelerate bone healing.

### Electronic supplementary material

Below is the link to the electronic supplementary material.

Supplementary Material 1: Additional data (Tables S1–S2, Figures S1–S21)

## Data Availability

All data needed to support the conclusions are present in the paper or the additional materials.
