# Supplementary material for: Injectable ultrasound-powered bone-adhesive nanocomposite hydrogel for electrically accelerated irregular bone defect healing
Source: J Nanobiotechnology. 2024 Feb 7;22:54. doi: 10.1186/s12951-024-02320-y (PMC10851493; doi:10.1186/s12951-024-02320-y)
Supplement: Supplementary file 1 — Supplementary Material 1: Additional data (Tables S1–S2, Figures S1–S21) [file 12951_2024_2320_MOESM1_ESM.docx]

*Electronic supplementary information*

**Injectable Ultrasound-Powered Bone-Adhesive Nanocomposite Hydrogel for Electrically Accelerated Irregular Bone Defect Healing**

Shiqi Zhou 1#, Cairong Xiao 2#, Lei Fan 4#, Jinghong Yang 1, Ruihan Ge 1, Min Cai 1, Kaiting Yuan 1, Changhao Li 1, Ross William Crawford 5, Yin Xiao 6, Peng Yu 2, Chunlin Deng 2, Chengyun Ning 2*, Lei Zhou 3*, Yan Wang 1*

*1* *Hospital of Stomatology, Guanghua School of Stomatology, Sun Yat-sen University, Guangdong Provincial Key Laboratory of Stomatology, Guangzhou, Guangdong, 510055, China*

*2 School of Materials Science and Engineering, National Engineering Research Center for Tissue Restoration and Reconstruction, South China University of Technology, Guangzhou, Guangdong, 510641, China*

*3* *Guangzhou Key Laboratory of Spine Disease Prevention and Treatment, Department of Spine Surgery, The Third Affiliated Hospital, Guangzhou Medical University, Guangzhou, Guangdong, 510150, China*

*4* *Department of Orthopedic Surgery, Nanfang Hospital, Southern Medical University, Guangzhou, Guangdong,* *510515, China*

*5 Institute of Health and Biomedical Innovation & Australia-China Centre for Tissue Engineering and Regenerative Medicine, Centre for Biomedical Technologies, Queensland University of Technology, Queensland 4059, Australia*

*6 School of Medicine and Dentistry & Menzies Health Institute Queensland, Griffith University, Queensland 4111, Australia*

*E-mail: imcyning@scut.edu.cn (C.Y.N.); zhoul@gzhmu.edu.cn (L.Z.);* *wangyan9@mail.sysu.edu.cn (Y.W.)*

** Corresponding Authors*

*# These authors contributed equally to this work*

**Table S1. Primer sequences of osteogenic differentiation markers of BMSCs**

| **Genes** | **Primers Sequences** |
| --- | --- |
| GAPDH | Forward: 5’-AAGAGGGATGCTGCCCTTAC-3’ |
|  | Reverse: 5’-CCAATACGGCCAAATCCGTTC-3’ |
| ALP | Forward: 5’-CCAACTCTTTTGTGCCAGAGA-3’ |
|  | Reverse: 5’-GGCTACATTGGTGTTGAGCTTTT-3’ |
| Runx2 | Forward: 5’-GGGAACCAAGAAGGCACAGA-3’ |
|  | Reverse: 5’-ACTTGGTGCAGAGTTCAGGG-3’ |
| BMP2 | Forward: 5’-TTCCATCACGAAGAAGCCGT-3’ |
|  | Reverse: 5’-GAAACTCGTCACTGGGGACA-3’ |
| OPN | Forward: 5’-TGGCTGAATTCTGAGGGACTAAC-3’ |
|  | Reverse: 5’-TTCTGAGATGGGTCAGGCAC-3’ |
| OCN | Forward: 5’-CCCTGAGTCTGACAAAGCCTTCA |
|  | Reverse: 5’-AGATGCGTTTGTAGGCGGTC-3’ |
| COL-1 | Forward: 5’-CACTGCAAGAACAGCGTAGC-3’ |
|  | Reverse: 5’-AGTTCCGGTGTGACTCGTG-3’ |

**Table S2. Primary and secondary antibodies applied in western blot**

| **Antibody** | **Catalogue Number** | **Manufacturer** |
| --- | --- | --- |
| GAPDH Mouse mAb | 60004-1-IG | Proteintech, America |
| Runx2 Rabbit pAb | 20700-1-AP | Proteintech, America |
| OCN Mouse mAb | sc-390877 | Santa Cruz, America |
| PI3K Rabbit pAb | ab191606 | Abcam, America |
| p-PI3K Rabbit pAb | ab182651 | Abcam, America |
| AKT Rabbit mAb | ab179463 | Abcam, America |
| p-AKT Rabbit pAb | ab38449 | Abcam, America |
| MEK1/2 Rabbit mAb | 8727T | Cell Signaling Technology, America |
| p-MEK1/2 Rabbit mAb | 9154T | Cell Signaling Technology, America |
| ERK1/2 Rabbit mAb | 4695T | Cell Signaling Technology, America |
| p-ERK1/2 Rabbit mAb | 4370T | Cell Signaling Technology, America |
| HRP Goat anti-Mouse IgG | 7076S | Cell Signaling Technology, America |
| HRP Goat anti-Rabbit IgG | 7074S | Cell Signaling Technology, America |


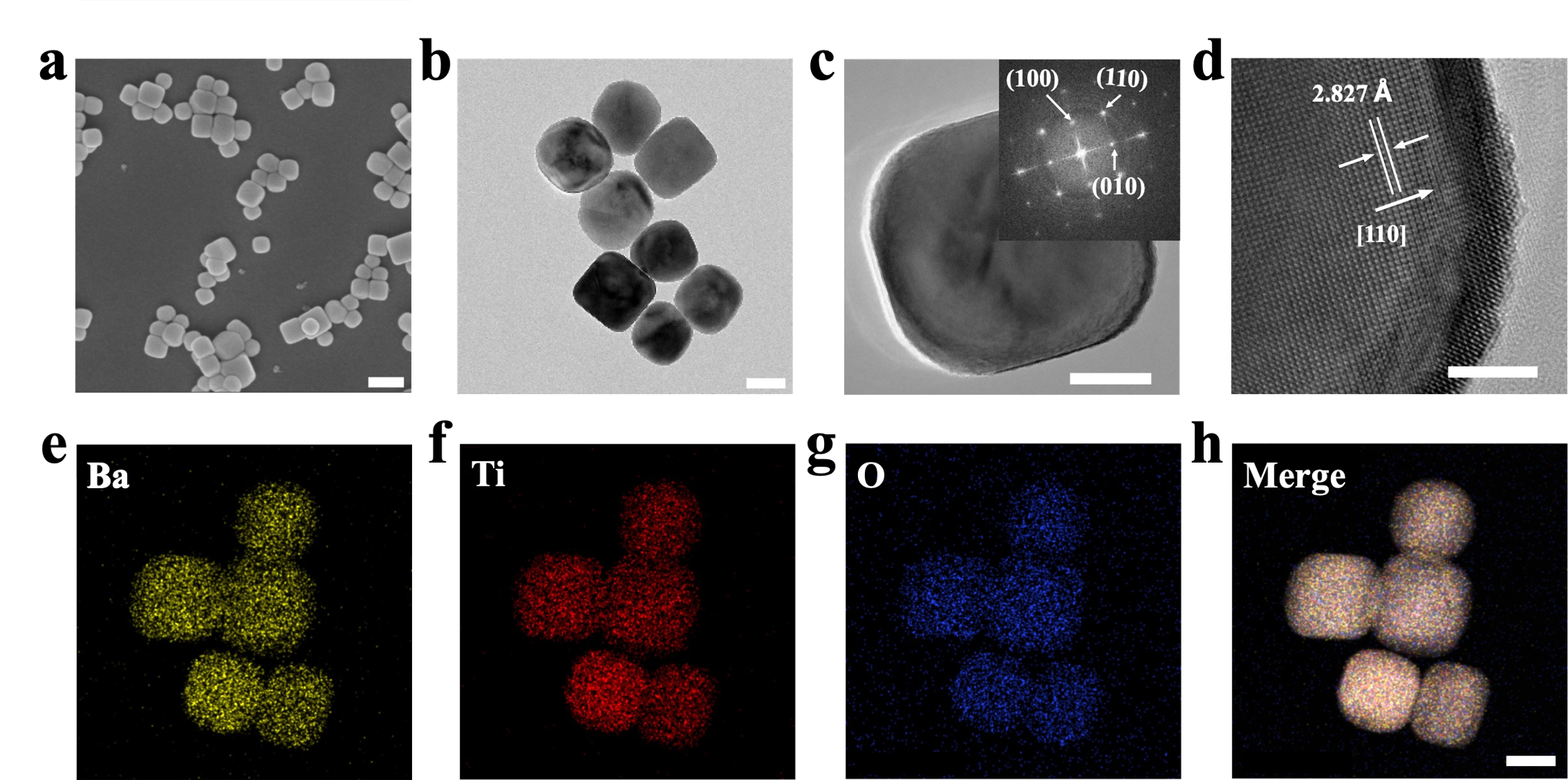


**Figure S1**. Characterization of KBTO nanoparticles. (a) SEM image. Scale bar represents 200 nm. (b, c) TEM images of KBTO nanoparticles with different magnification image and the corresponding selected area electron diffraction (SAED) pattern (insert). Scale bars represent 50 nm (left) and 20 nm (right). (d) High-resolution transmission electron microscope (HRTEM) image. Scale bar represents 5 nm. (e-h) EDS image shows the corresponding element mappings of Ba, Ti, and O. Scale bar represents 50 nm.


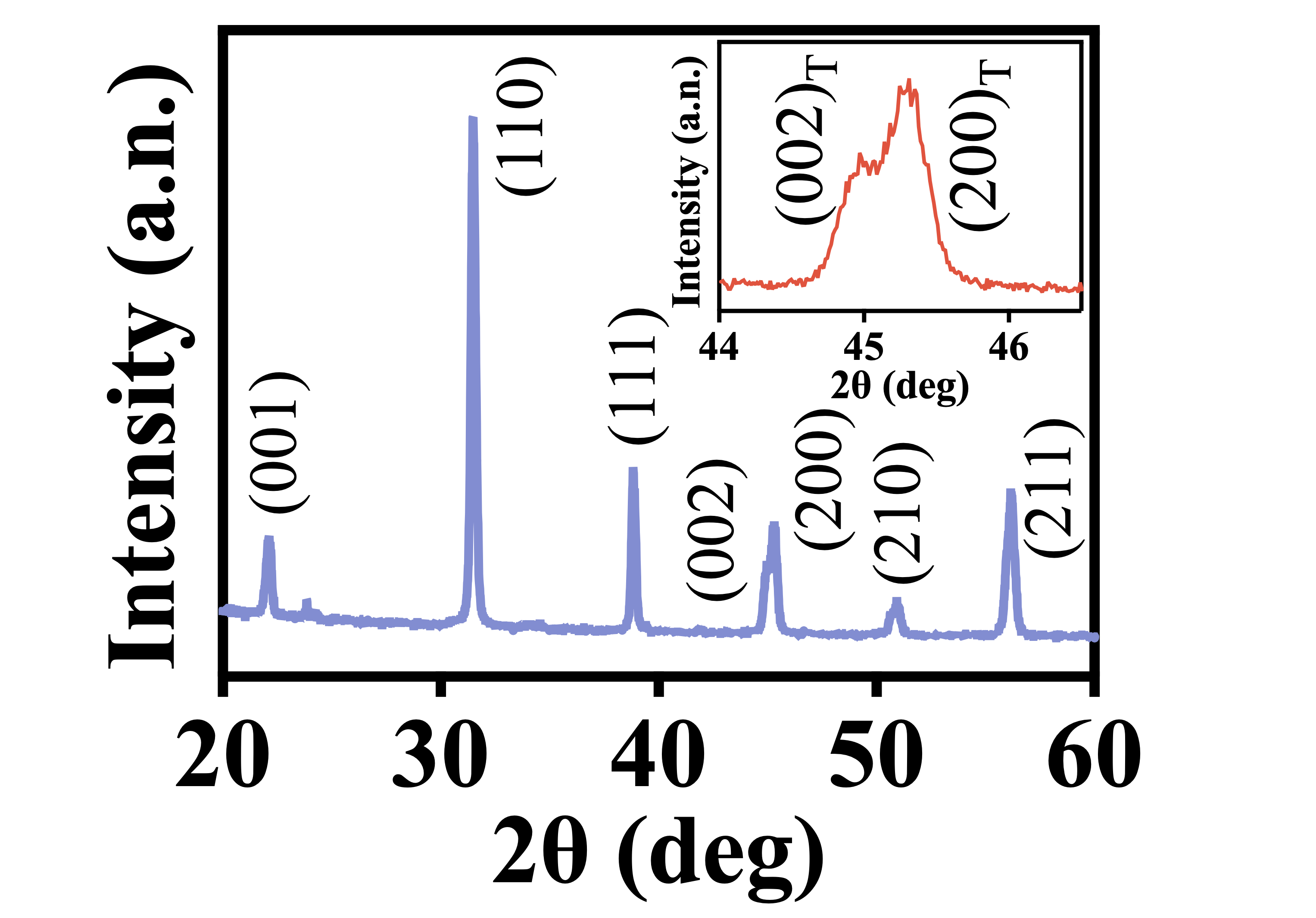


**Figure S2**. XRD pattern of KBTO nanoparticles and the peak splitting at around 2θ = 45° (insert).


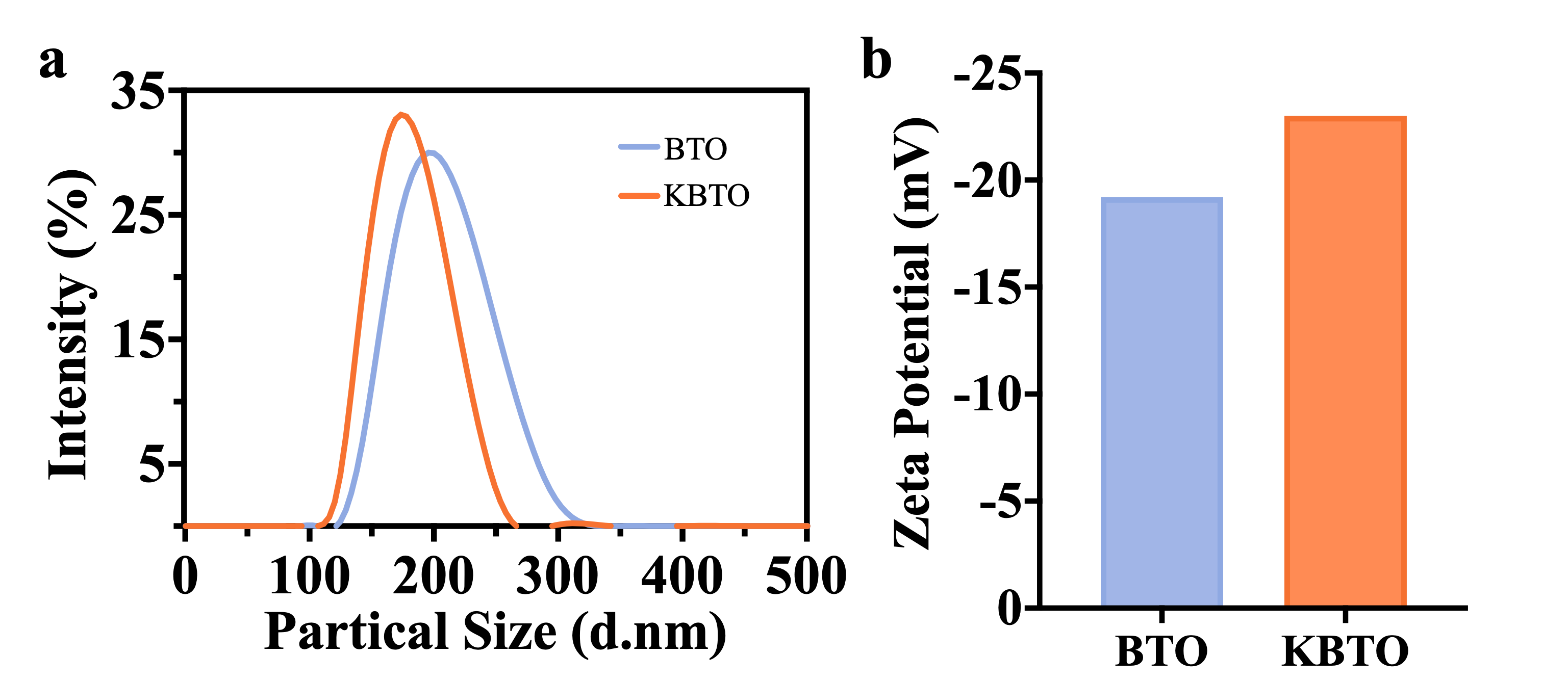


**Figure S3**. (a) Size distributions and (b) Zeta potentials of BTO and KBTO nanoparticles.


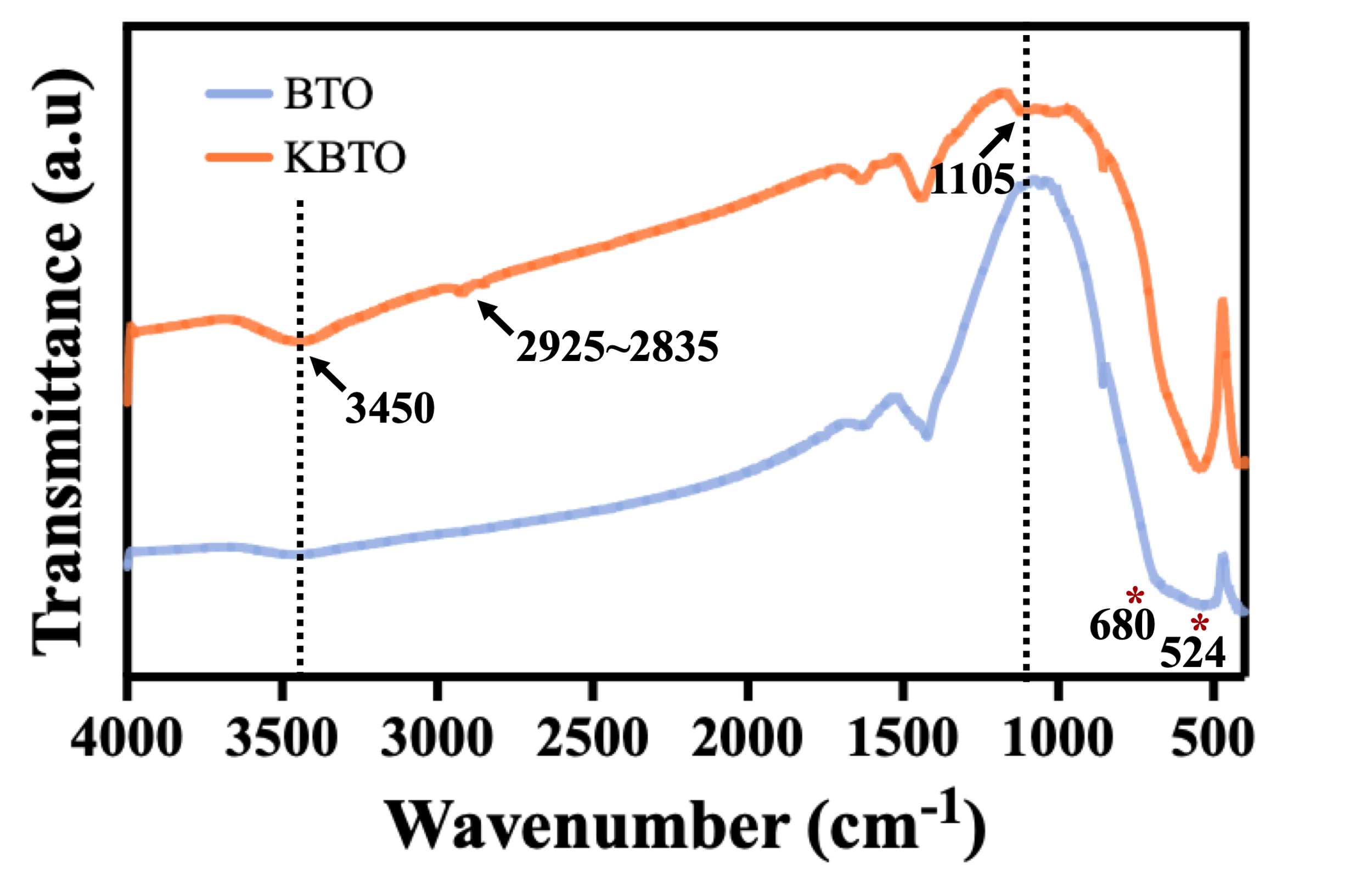


**Figure S4**. FTIR spectra of BTO and KBTO nanoparticles.

**Figure S5**. ^1^H NMR spectra of CS and OCS.


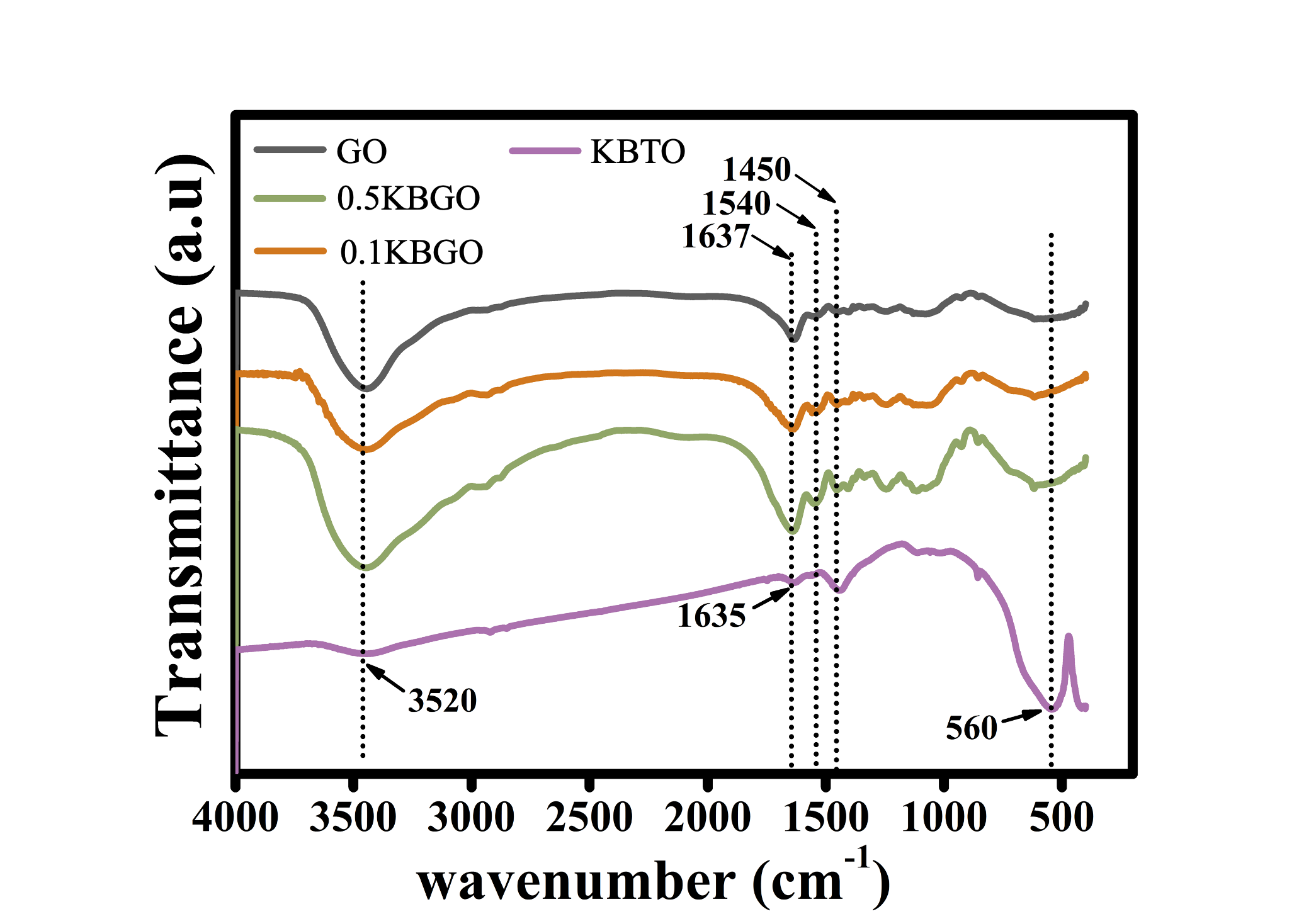


**Figur****e S6**. FTIR spectra of different hydrogels and KBTO nanoparticles.


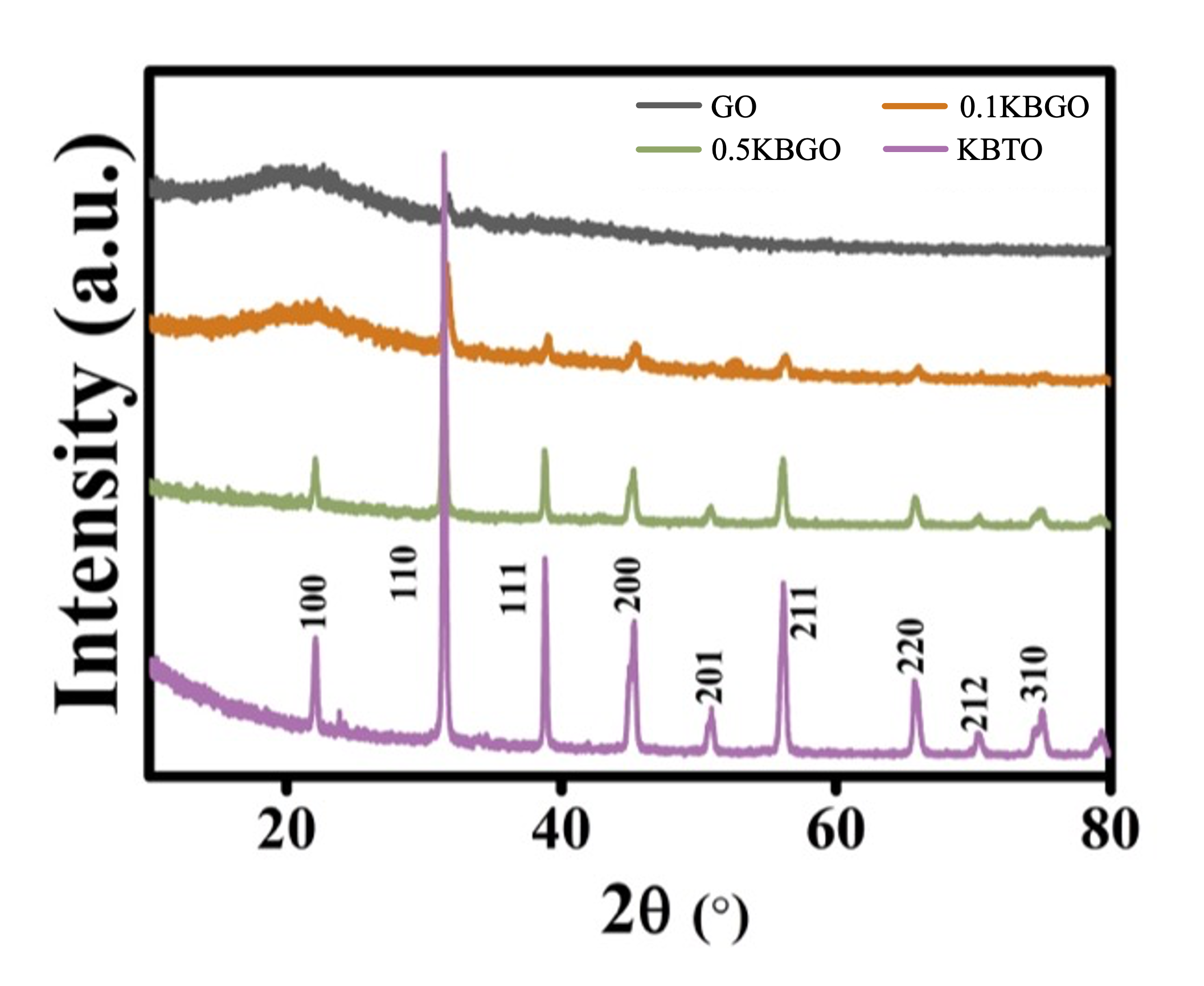


**Figure S7**. XRD spectra of different hydrogels and KBTO nanoparticles.


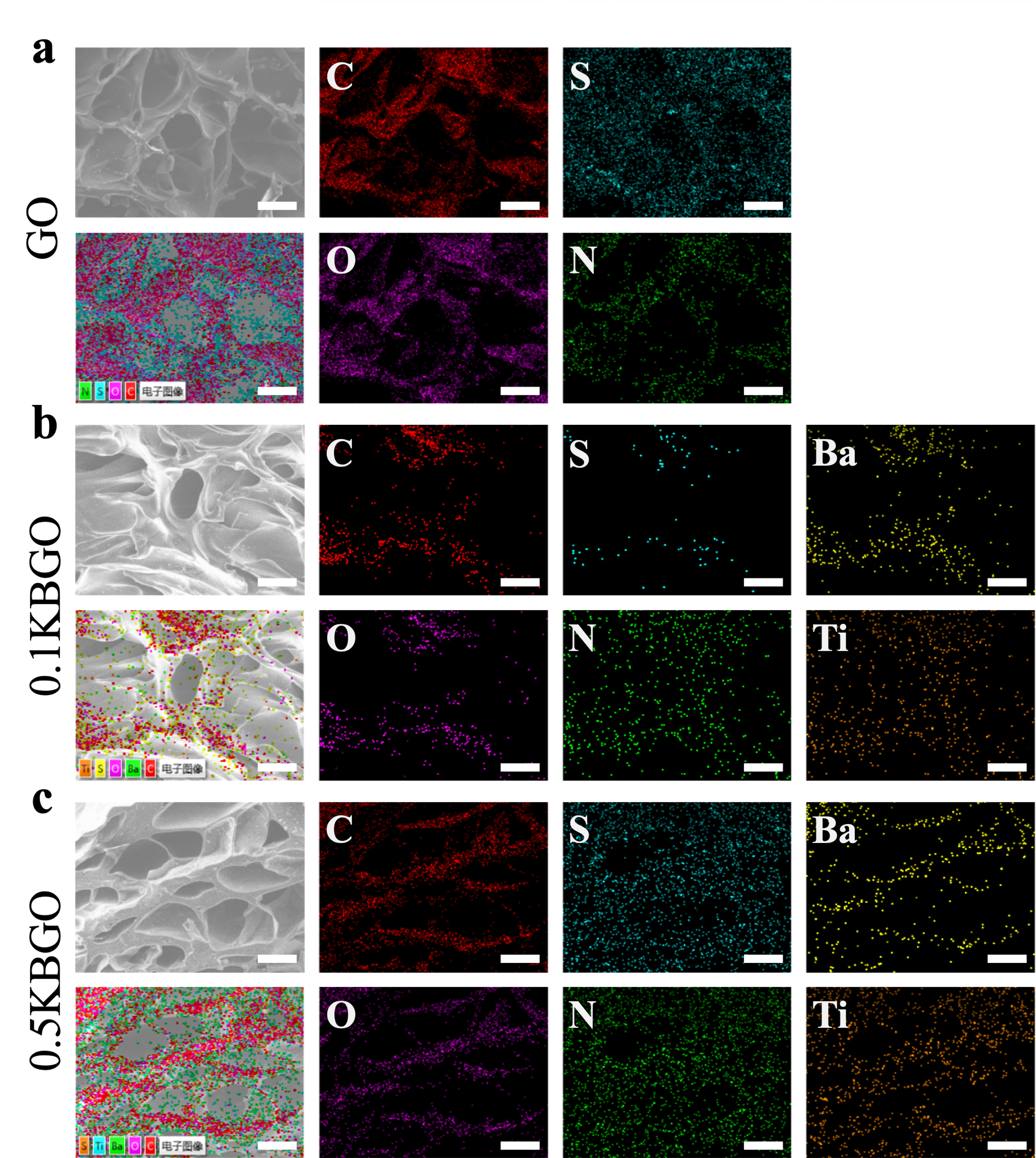


**Figure S8**. EDS analysis of the elements in GO, 0.1KBGO, and 0.5KBGO hydrogels. Scale bar represents 100 μm.


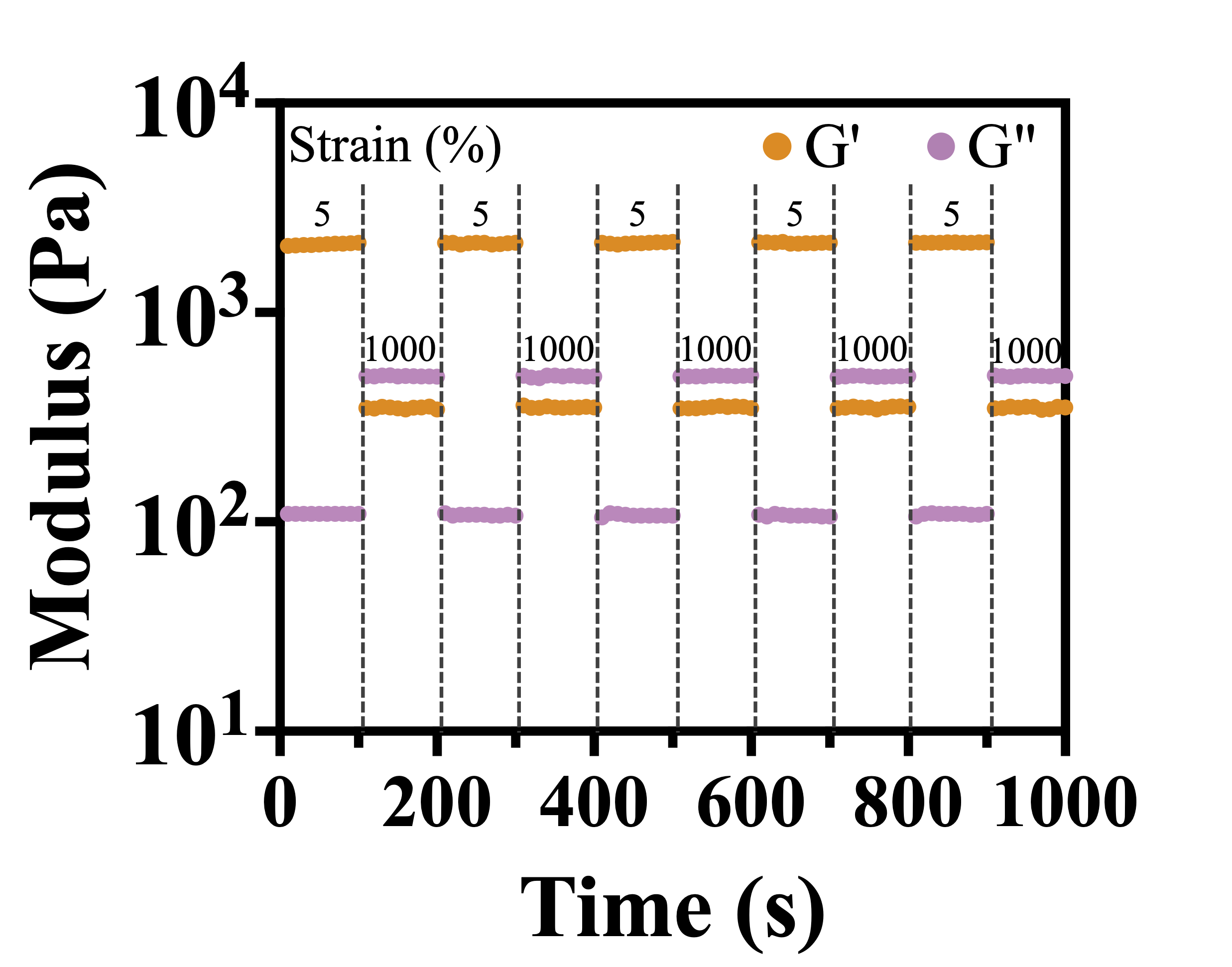


**Figure S9**. Rheological behaviors of 0.1KBGO hydrogel with alternate strains switched from 5% to 1000% for five cycles.

**Figure S10**. Stress–strain curves of the original (black line) and self-healed (red line) 0.1KBGO hydrogel.


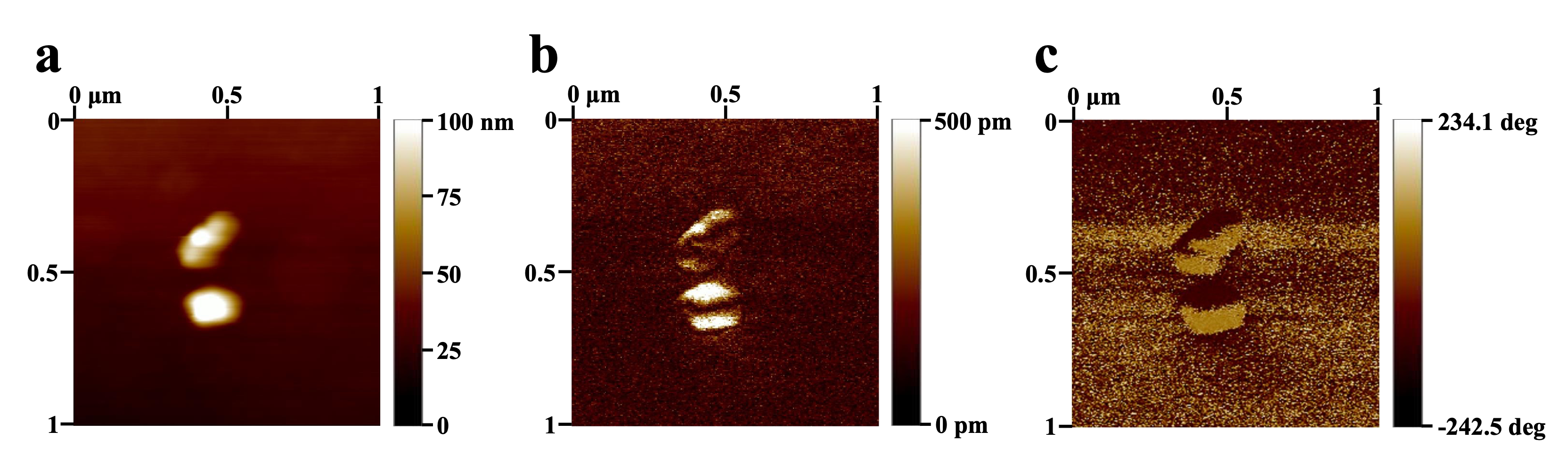


**Figure S11**. (a) AFM images of KBTO nanoparticles. The PFM images of KBTO nanoparticles for (b) amplitude and (c) phase.


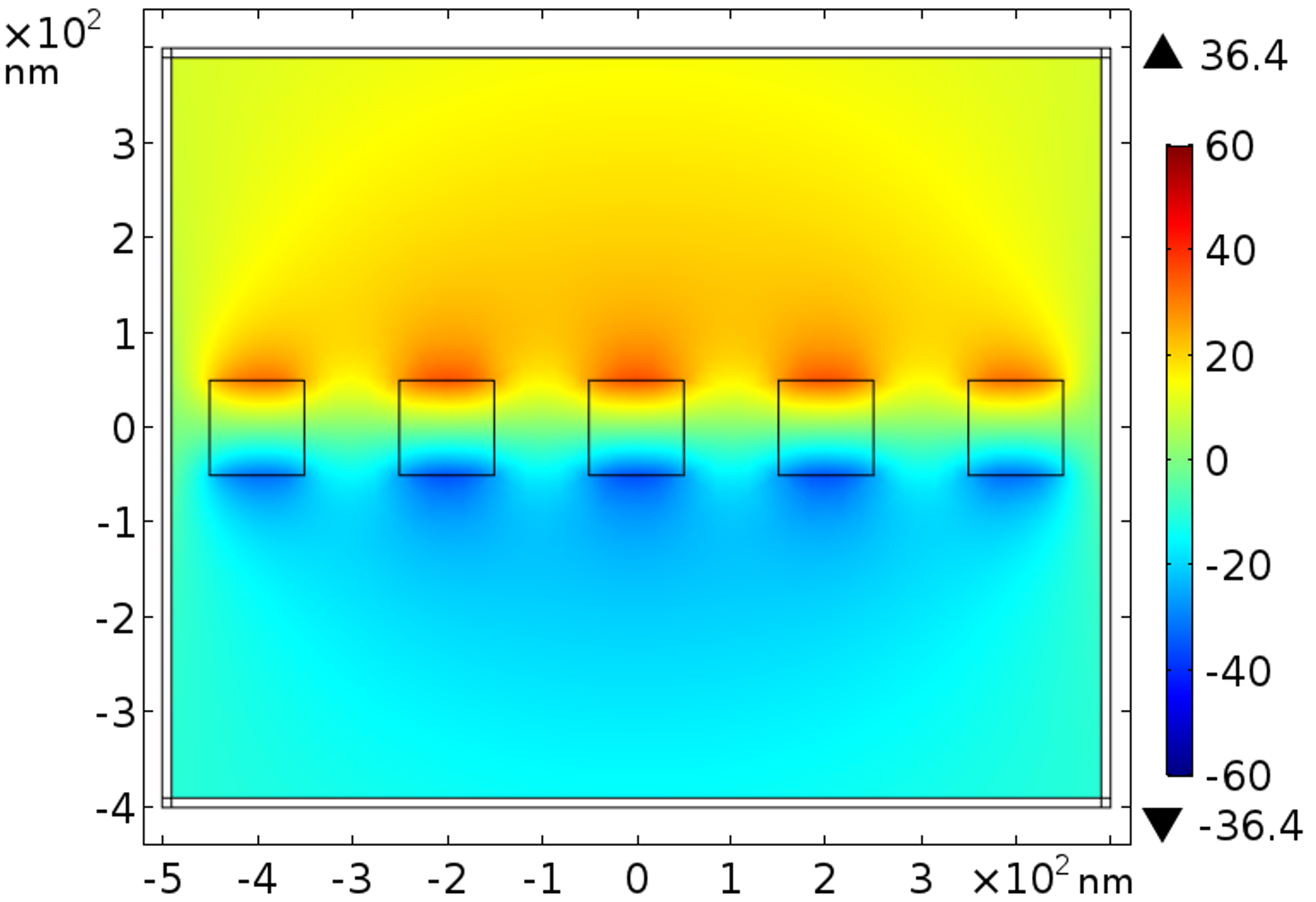


**Figure S12**. Finite-element simulation of the potential distribution of 0.1KBGO hydrogel under the pressure of 0.5*10^8^ Pa.

**
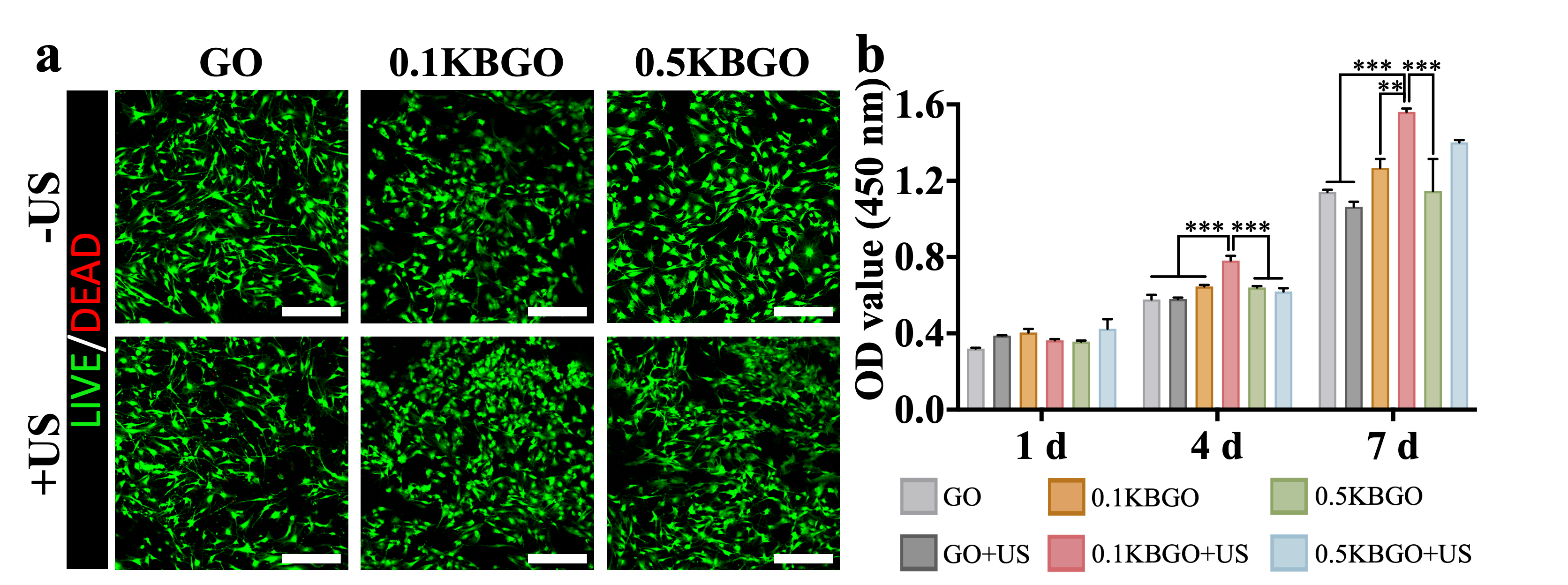
**

**Figure S13**. (a) Live/Dead staining of BMSCs incubated on each sample surface for 48 h. Scale bar represents 300 μm. (b) CCK-8 assay results of BMSCs cultured on different samples for 1, 4, and 7 days. ANOVA followed by Tukey’s post hoc test was performed for statistical analysis (**p* < 0.05, ***p* < 0.01, ****p* < 0.001).


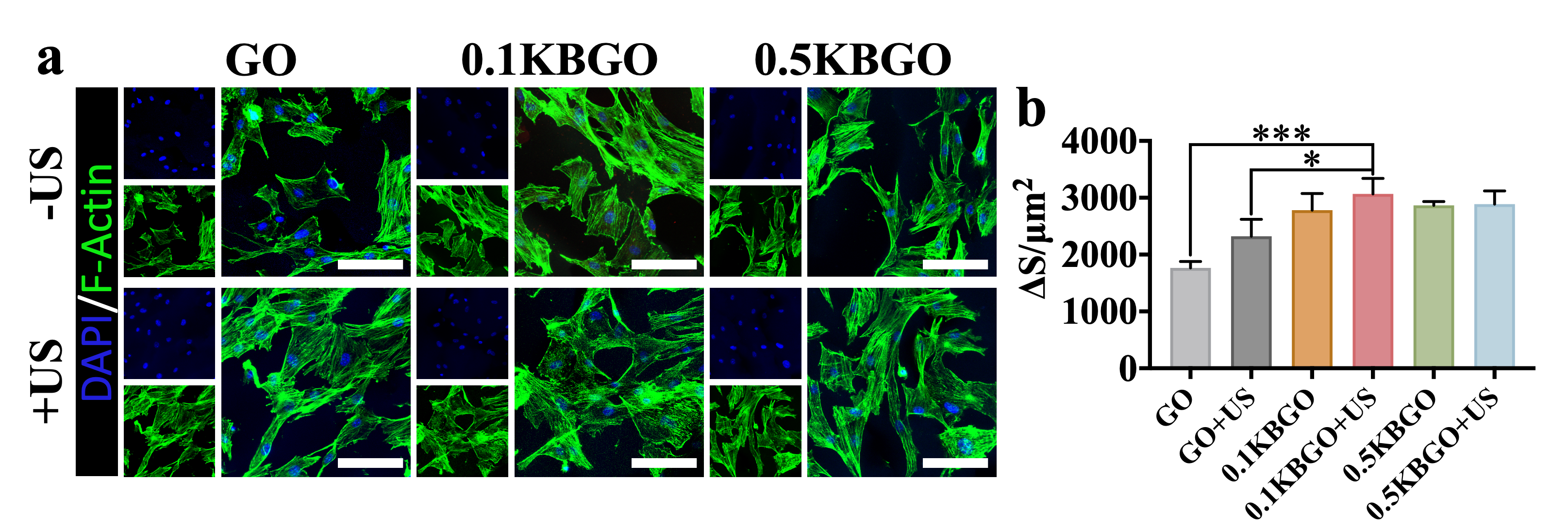


**Figure S14**. (a) Immunofluorescence images show the cytoskeleton (green) and nuclei (blue) of BMSCs cultured on each hydrogel for 48 h. Scale bar represents 100 μm. (b) Quantitative analysis of cell spread area. ANOVA followed by Tukey’s post hoc test was performed for statistical analysis (**p* < 0.05, ***p* < 0.01, ****p* < 0.001).


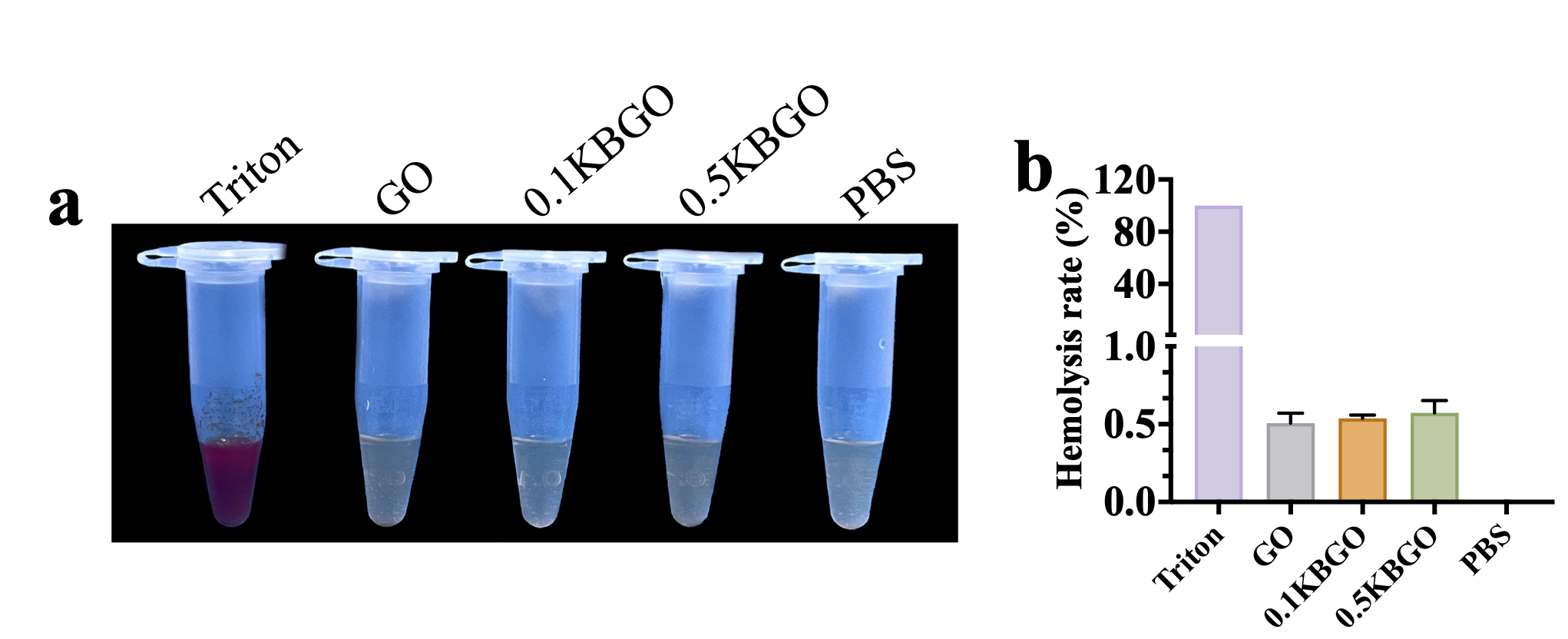


**Figure S15**. Assessment of hemocompatibility. (a) Images of blood compatibility. Triton was the positive control and PBS was the negative control. (b) Quantitative analysis of hemolysis rates in different groups. ANOVA followed by Tukey’s post hoc test was performed for statistical analysis (**p* < 0.05, ***p* < 0.01, ****p* < 0.001).


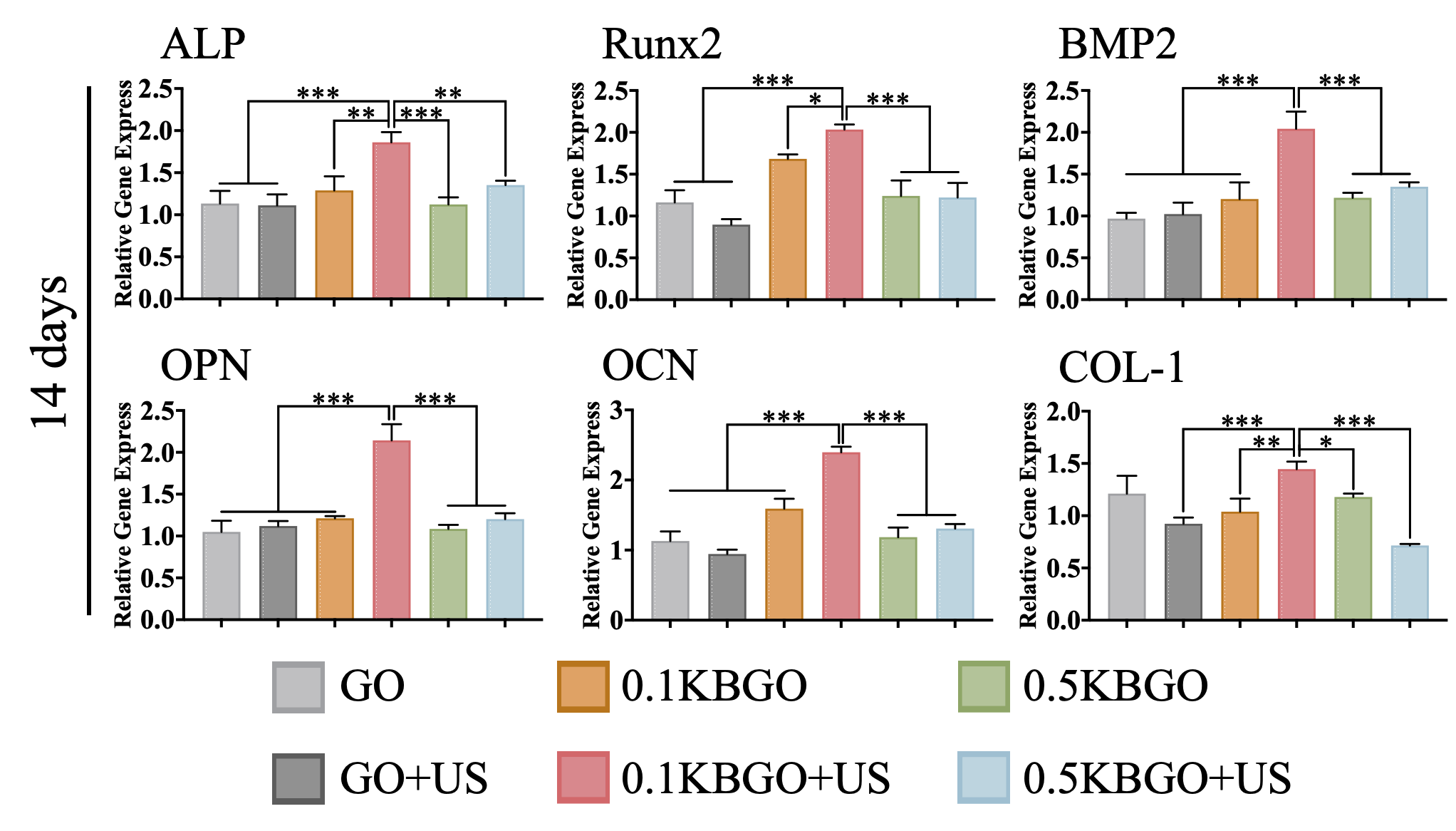


**Figure S16**. The expression levels of osteogenic genes (ALP, Runx2, BMP-2, OCN, OPN, and COL-1) of BMSCs cultured on different samples for 14 days. ANOVA followed by Tukey’s post hoc test was performed for statistical analysis (**p* < 0.05, ***p* < 0.01, ****p* < 0.001).


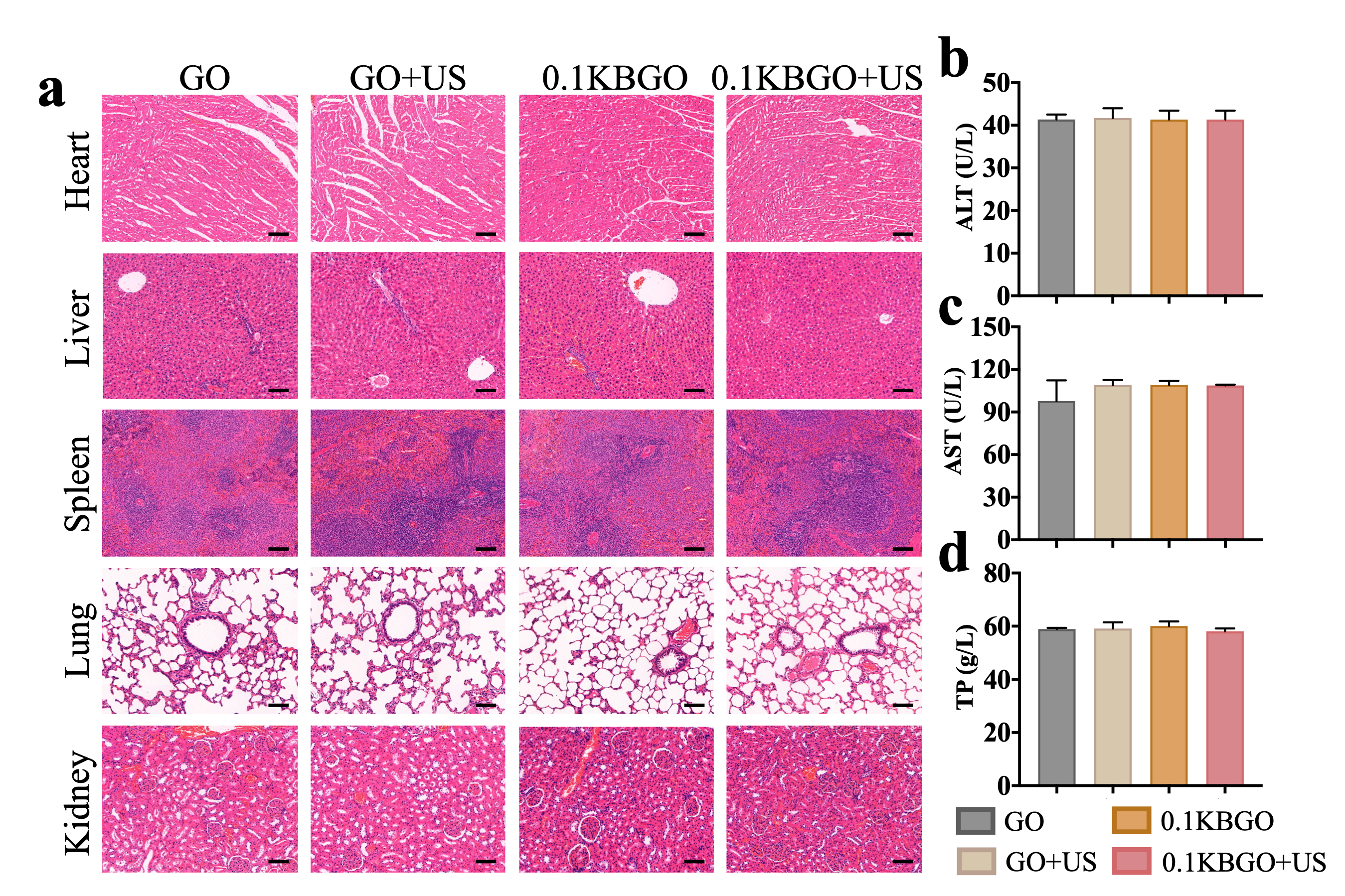


**Figure S17**. *In vivo* biocompatibility of ultrasound-powered bone-adhesive hydrogel. (a) H&E staining of heart, liver, spleen, lung, and kidney tissues sections from rats of different groups. Scale bar represents 100 μm. (b-d) The levels of ALT, AST, and TP in serum from rats of different groups. ANOVA followed by Tukey’s post hoc test was performed for statistical analysis (**p* < 0.05, ***p* < 0.01, ****p* < 0.001).


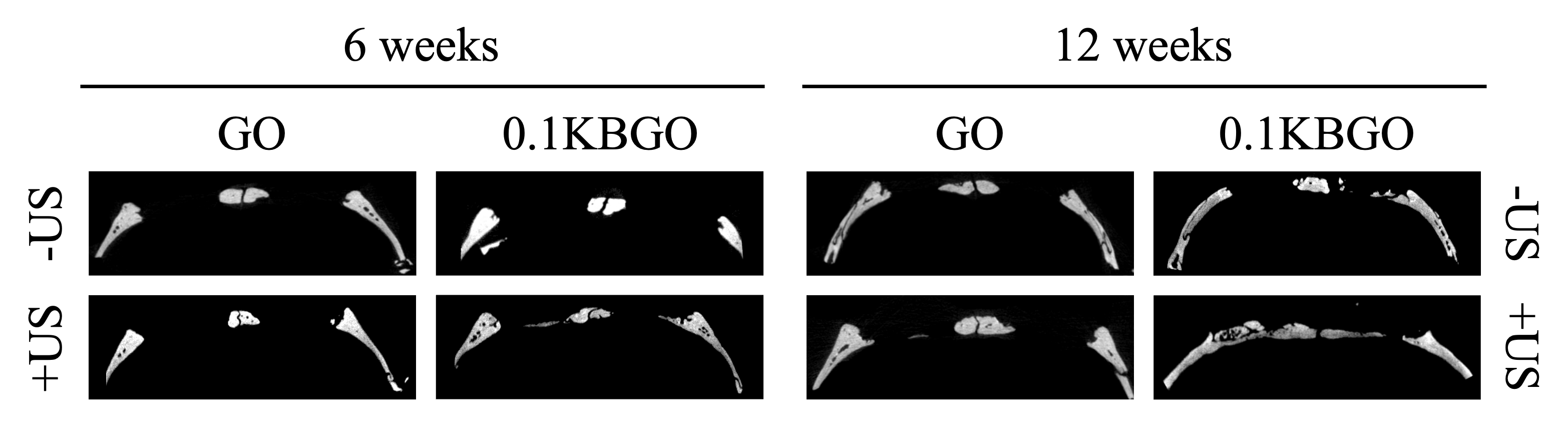


**Figure S18**. Micro-CT images showing sagittal plane bone regeneration in different groups at weeks 6 and 12 postoperatively.


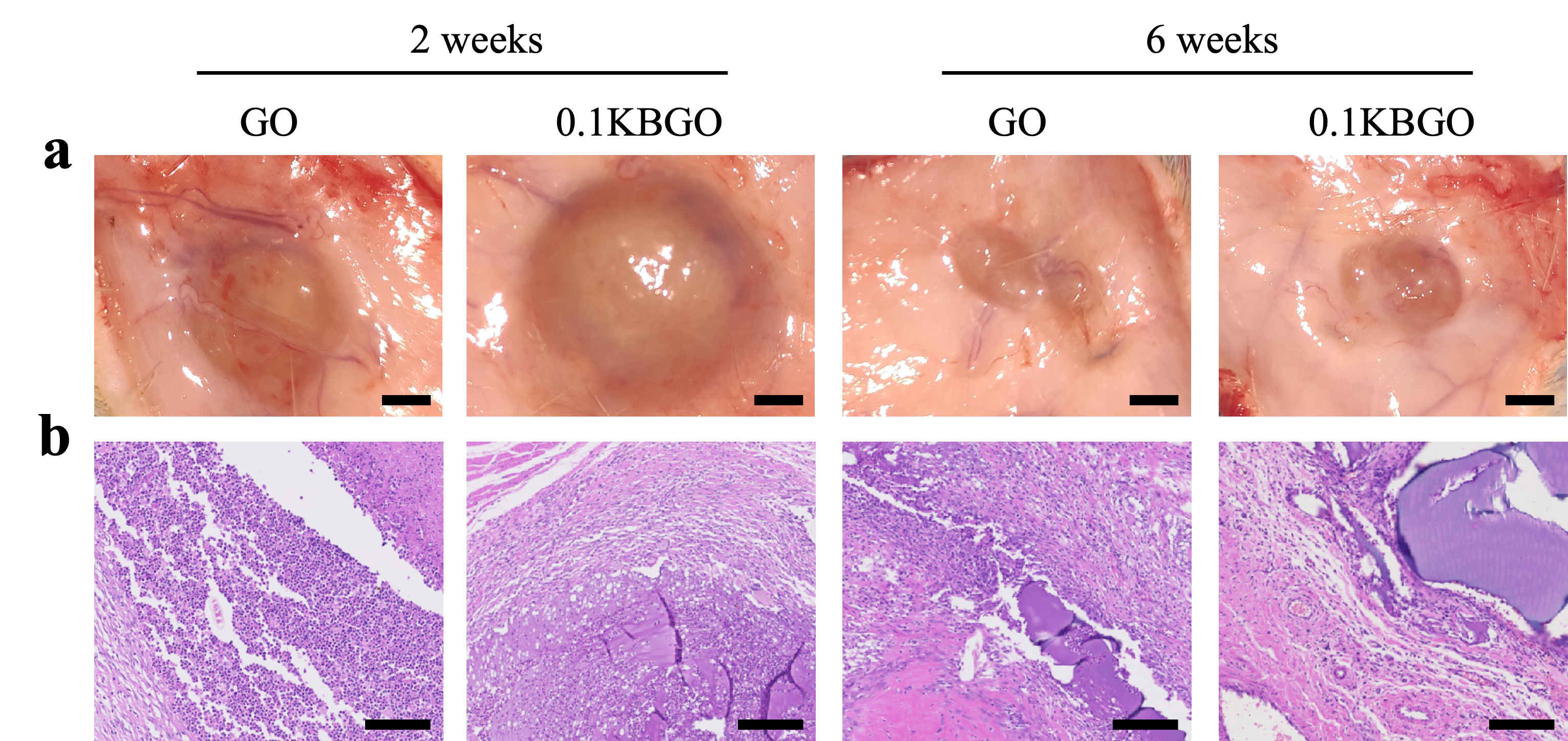


**Figure S19**. *In vivo* biodegradation of implanted hydrogels. (a) Optical images. Scale bar represents 200 mm. (b) H&E staining. Scale bar represents 100 μm；


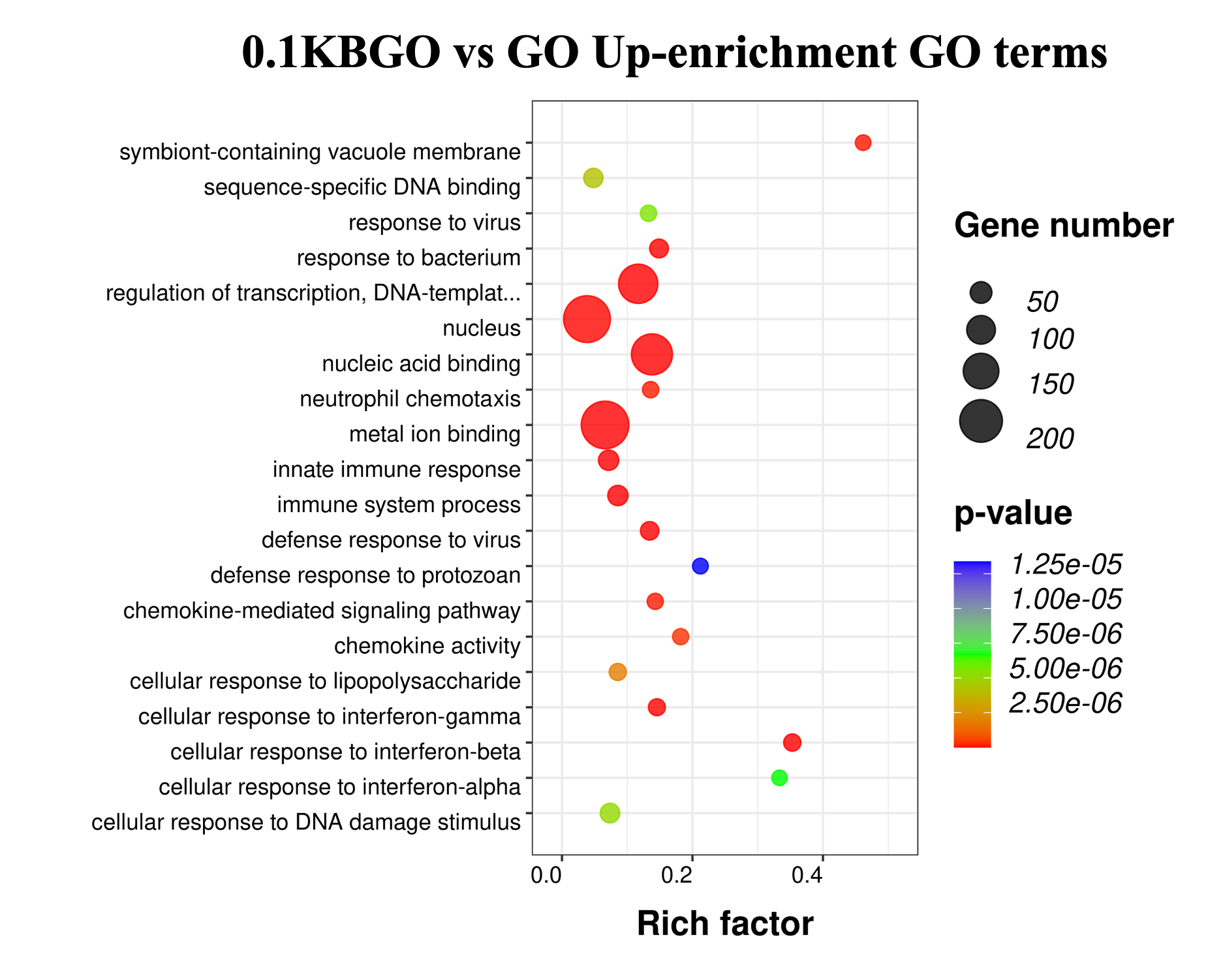


**Figure S20**. The top 20 significant up-enrichment GO terms of 0.1KBGO *vs* GO.


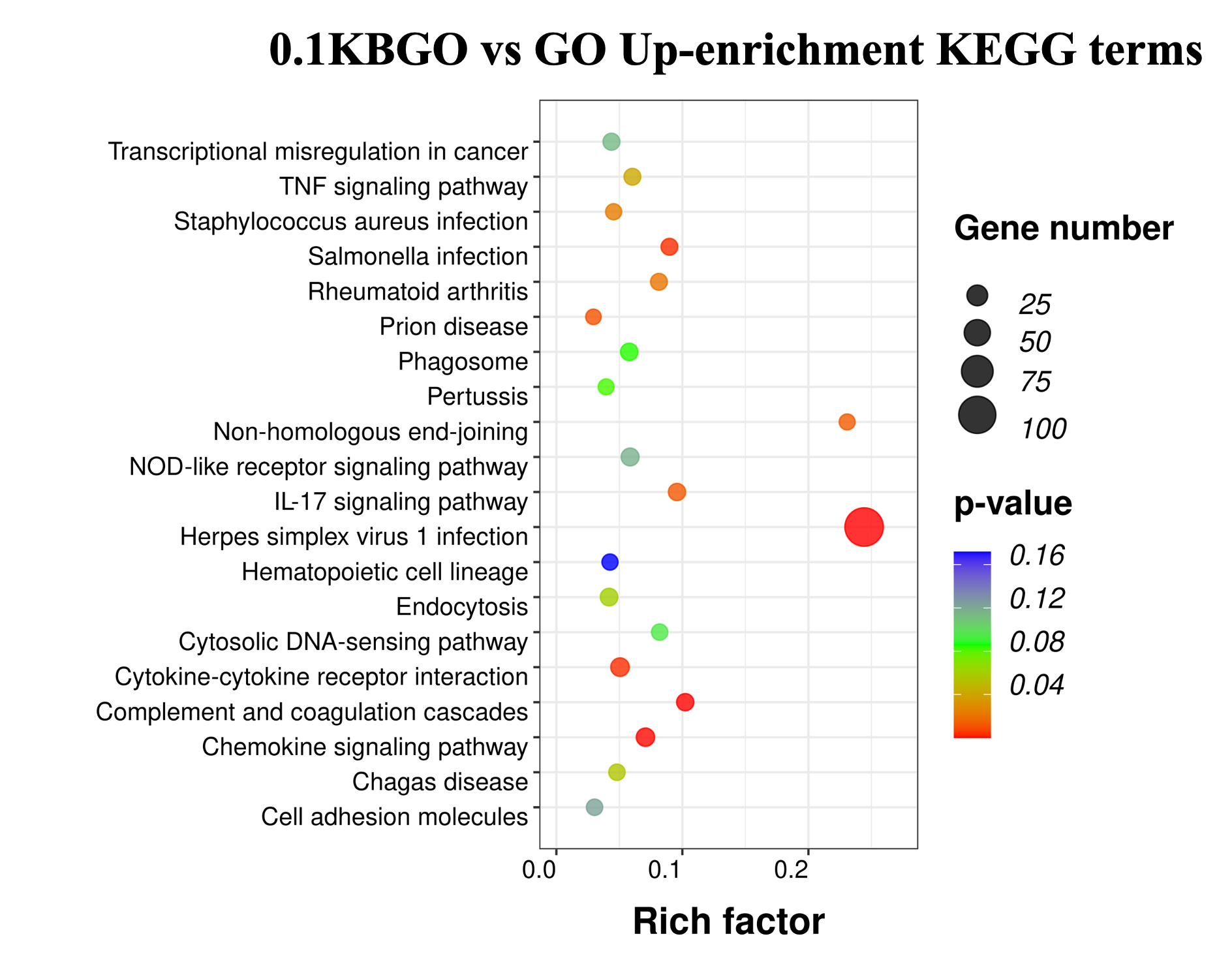


**Figure S21**. The top 20 significant up-enrichment KEGG pathways of 0.1KBGO *vs* GO.
